# Electrically responsive multilayer soft actuators using a solvent-free high dielectric permittivity polysiloxane ink

**DOI:** 10.1039/d6mh00302h

**Published:** 2026-04-01

**Authors:** Jana Wolf, Patrick M. Danner, Thulasinath Raman Verkatesan, Valentin Razvan Lupu, Dorina M. Opris

**Affiliations:** a Swiss Federal Laboratories for Materials Science and Technology – Empa, Laboratory for Functional Polymers Ueberlandstr. 129 Dübendorf CH-8600 Switzerland dorina.opris@empa.ch; b Department of Materials, ETH Zürich Vladimir-Prelog-Weg 5 Zürich CH-8093 Switzerland

## Abstract

Multilayer soft dielectric elastomer actuators (DEAs) consist of stacked elastic capacitors that convert electrical energy into mechanical work. The generated mechanical work and force can be increased by reducing the dielectric layer thickness, increasing the material's dielectric permittivity, or increasing the number of layers. Despite major progress in developing high-permittivity elastomers, integrating these materials into multilayer devices remains challenging. To date, large-scale production of stack DEAs has been achieved only with commercial polydimethylsiloxane (PDMS), which has a low dielectric permittivity of about 3. Here, we report a solvent-free, high-dielectric-permittivity capillary ink that combines a pot life of more than 300 days at −30 °C, good processability comparable to that of commercial PDMS, and rapid thermal crosslinking, fulfilling essential criteria for the industrial-scale fabrication of stack actuators. The ink wets a preheated metal substrate at 100 °C to form highly uniform ultrathin films that cross-link in just 2 minutes. The resulting elastomer exhibits a dielectric permittivity of 11 at 10 Hz, a storage modulus of 350 kPa, and negligible mechanical losses. Single-layer circular DEAs exhibit a 5.7% lateral strain at 26.2 V µm^−1^ and a stable actuation over 5000 cycles at 24.7 V µm^−1^. At an electric field of 19.0 V µm^−1^, a stripe actuator exhibits 5.5% lateral strain and 1 Hz, which increased to 9% at 5 Hz. The actuators respond to a low voltage of 500 V, corresponding to 25 V µm^−1^, and provide fast, reversible actuation with a displacement of 25 µm. These results demonstrate a scalable route to high-performance DEAs, marking a significant step towards the industrial application of high-dielectric permittivity polysiloxanes.

## Abstract 2

Multilayer soft dielectric elastomer actuators (DEAs) consist of stacked elastic capacitors that convert electrical energy into mechanical work.

## Graphical abstract

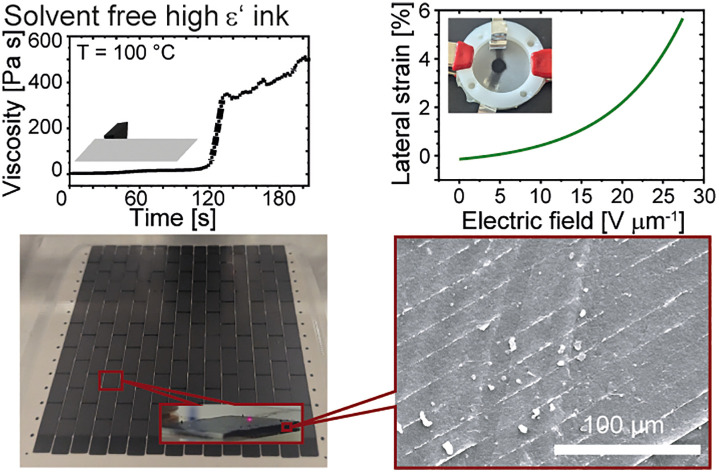

**New concepts**

Although high dielectric permittivity polysiloxanes have been extensively developed for soft actuators, their integration into multilayer stacks remains impractical due to processing limitations, including dewetting at dielectric–substrate and electrode–dielectric interfaces and reliance on solvent-based thin-film processing. This compromises the stack's homogeneity and can lead to dielectric actuator failure. Consequently, industrial production of stack actuators still relies on polydimethylsiloxane (PDMS), which has a low dielectric permittivity, necessitating operation at voltages above 1 kV. Here, we have synthesized a solvent-free capillary ink that prevents dewetting and enables the formation of highly uniform, mechanically compliant dielectric films compatible with the electrode. Unlike solvent-based strategies, which are incompatible with multilayer manufacturing, our ink enables uniform thin-film formation directly on metal substrates without solvent use. Additionally, it enables rapid crosslinking within 2 minutes at 100 °C, while maintaining a long pot life. The resulting elastomer exhibits a low elastic modulus (350 kPa), negligible mechanical losses, and a high dielectric permittivity. These enabled scalable fabrication of 20-layer dielectric stacks (20 µm per layer) that reliably and reversibly actuate at 500 V, advancing sustainable, low-voltage soft-actuator technologies. This is the first time a high-dielectric, soft, functional material has been used in the scalable manufacturing of multilayer actuators.

## Introduction

Dielectric elastomer actuators (DEAs) are soft, electroactive materials that convert electrical energy into mechanical work. Owing to their high compliance, large strain, and rapid response, DEAs are often referred to as artificial muscles. These characteristics position them as promising candidates for emerging applications, including soft robotics, haptic interfaces, reconfigurable optics, and adaptive surfaces.^1–5^

A DEA is essentially an elastic capacitor, consisting of a thin, dielectric elastomer layer sandwiched between two compliant electrodes.^6–9^ When a voltage, typically in the kilovolt range, is applied to the compliant electrodes, the oppositely charged electrodes attract each other, generating an electrostatic pressure referred to as Maxwell pressure (*p*, eqn (1)):

1 *p* = *ε*_0_*ε*_r_*E*^2^

where *ε*_0_ is the vacuum permittivity, *ε*_r_ is the relative dielectric permittivity of the material, and *E = Ud*^−1^ is the applied electric field.^10–14^ Therefore, the Maxwell pressure is directly proportional to the dielectric permittivity and the square of the electric field. Consequently, high Maxwell pressures can be achieved using thin elastomer layers and high-dielectric-permittivity materials. This pressure causes the elastomer to compress in the thickness direction.^15–21^ Since the elastomer is nearly incompressible with a Poisson's ratio of 0.5, it consequently expands in plane.^22–24^ When the capacitor is discharged, the elastic restoring force brings it back to its original shape. Appropriately cross-linked dielectric elastomers exhibit fast, reversible deformation.^15^

The thickness actuation strain (*s*_*z*_) can be predicted by eqn (2). It is inversely proportional to *Y* and directly proportional to *ε*_r_ and the square of the electric field.^16,24^2
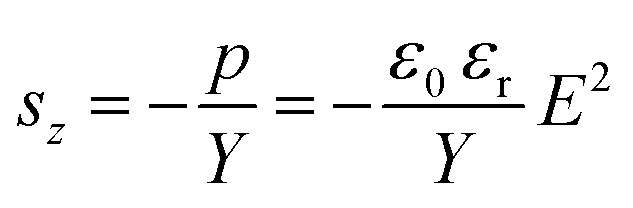
The electrodes are crucial to the actuator's functionality. They must be compliant to allow repeated large deformations of the elastomer without losing their integrity, and they must adhere to the dielectric to prevent delamination. Furthermore, they should not significantly affect the elastomer's stiffness.^12,25,26^ Self-clearing can affect the electrodes' lifespan, making the actuators more fault-tolerant.^27,28^ Commonly used electrode materials include carbon grease,^2,11,16,29^ carbon powder,^25,30^ carbon nanotubes (CNTs, including single-walled (SWCNT) and multi-walled (MWCNT)),^4,14,29,31^ thin metal films (*e.g.* silver),^32^ conductive composites,^33^ graphite nanoplatelets,^34,35^ and liquid metals like EGaIn.^36^

An ideal elastomer should exhibit high dielectric permittivity, high breakdown strength, and a low Young's modulus.^6,18,28^ The low Young's modulus allows the actuator to be easily deformed by the Maxwell pressure, while the high dielectric permittivity elastomers allow for storing more charges. However, most elastomers have a low dielectric permittivity, typically 3–6. Strategies to enhance permittivity include incorporating highly polarizable fillers into the polymer matrix^37^ or introducing polar functional groups along the polymer backbone.^38–45^ The mechanical losses and creep affect the frequency response and reliability of DEAs, leading to hysteresis between cycles. A drop in actuation strain is often observed at higher frequencies because the elastomer does not have sufficient time to fully recover its initial shape after the electric field is removed.^17,27,46^ In addition, the dielectric film should be thin, since the actuation strain is inversely proportional to the film thickness. To integrate a dielectric material into a stack, the ink must be solvent-free, as the presence of solvent interferes with the underlying layers and causes defects. However, achieving a solvent-free, processable material with a high dielectric permittivity while maintaining favorable mechanical and electromechanical properties remains a significant challenge.^45,47^

Commonly used materials include acrylates, nitrile butadiene rubber (NBR), polyurethanes, and silicones.^9,17,26,33^ Acrylic elastomers (*e.g.* 3M VHB-4910/VHB-4905J) have a dielectric permittivity of 4.7 and withstand high electric fields, leading to high actuation strains. However, they often require prestreaching and thus a rigid frame to keep the prestrain, suffer from viscoelastic losses, and show creep and hysteresis.^1,7,17,24,48,49^ Additionally, they have a much higher glass transition temperature (*T*_g_) than polysiloxanes, which limits their use at low temperatures and high frequencies. NBR offers higher dielectric permittivity, higher *Y*, better stress relaxation, and greater energy density than some commercial dielectric elastomers. NBR can be tuned for fast cross-linking, and its mechanical properties are easily adjustable, making it a promising material for stack actuators.^9,50^ Also, these materials have a *T*_g_ close to 0 °C, which limits their use in outdoor applications.

Currently, one of the most promising material classes is based on silicone elastomers (*e.g.*, PDMS, Wacker Elastosil®), which exhibit lower viscoelastic losses than acrylates, enabling faster actuation.^8^ Their inherent dielectric permittivity is typically lower than that of acrylates (*e.g.*, 2.8), which results in lower Maxwell pressure. However, recent advancements in polysiloxanes (silicones) have achieved high dielectric permittivity through chemical modification, along with low *T*_*g*_ for flexibility at room temperature.^43^ Silicones can be formulated for 3D printing, exhibit low Young's moduli, and show good electromechanical stability, providing a good balance between elasticity and durability, which is attractive for healthcare device applications and biomimetic soft robotic applications.^2,16,51–54^

Despite their promise, the practical implementation of DEAs close to or in the human body remains limited by a key constraint: the need for high driving voltages, caused by the use of a low-dielectric-permittivity.^29,55–57^ While reducing the thickness of the dielectric film is an effective way to lower the driving voltage, it remains extremely challenging to manufacture large, defect-free films with precise thicknesses that can withstand very high electric fields.^11,28,45^

DEAs are susceptible to various breakdown mechanisms, such as electromechanical instability (EMI), which occurs when the elastomer undergoes progressively greater strain, becoming thinner and thinner until failure.^3,28^ Additionally, electrothermal breakdown resulting from localized resistive heating at a “hot spot” can cause dielectric puncture (pin-hole formation) or membrane rupture. Due to their thermal insulation, multilayer DEAs are more prone to overheating, leading to dielectric breakdown.

Multilayer DEAs give a linear increase in displacement (stroke) and force output in the thickness direction with increasing the number of layers.^23,58–60^ To achieve large actuation in stack DEAs at a given applied voltage, dielectric layers with a thickness below 50 µm should be manufactured.^6,12,28,61^ Numerous manufacturing processes for stack DEAs have been reported, including manual stacking,^44^ folding,^62,63^ automated thin-film processing,^23^ casting,^64,65^ spray deposition,^23,57^ slot-die coating,^50^ and 3D printing.^16^ However, traditional stacking processes are often time-consuming, generate waste, and result in non-uniform dielectric layers, thereby reducing yield.^3,66^ In addition, the stack layers may suffer from delamination, which is best mitigated using a wet layer-by-layer fabrication approach. Thus, the manufacturing process involved in their production is difficult to scale up.^18,29^

We recently introduced a new class of high dielectric permittivity polysiloxanes modified with different types and contents of polar groups, combining a low *T*_g_ with a high *ε*_r_.^43^ Among the various polar functional groups evaluated, the nitrile group proved to be the most promising.^43,67^ However, polar polysiloxanes present significant processing challenges, and the mechanical performance of the resulting thin elastomer films remains suboptimal.^68^ Notably, dewetting on substrates is frequently observed^13^ and the elastomers often exhibit substantial viscous losses.^47,69^ These limitations have hindered the use of polar polysiloxane-based elastomers in industrial DEA production.

Here, we developed a solvent-free capillary ink^70^ comprising a commercial PDMS formulation and a nitrile-functionalized polysiloxane. The ink can be processed into a uniform thin film on industrial substrates such as stainless steel, thereby solving several processing limitations of stacked DEAs. The resulting elastomer exhibits a dielectric permittivity more than three times that of conventional PDMS, along with a reduced Young's modulus of 350 kPa. Furthermore, the ink was successfully used to manufacture stack actuators with a dielectric thickness of 20 µm that respond to low voltages. These combined properties position our material as a promising candidate for the large-scale production of stack DEAs.

## Experimental

### Materials

Karstedt's catalyst (2.1–2.4% platinum divinyltetramethyldisiloxane in xylene) was purchased from ABCR. 3-Cyanopropylmethyldichlorosilane was purchased from Gelest. 2,4,6,8-Tetramethyl-2,4,6,8-tetrakis({3-[(oxiran-2-yl)methoxy]propyl})-1,3,5,7,2,4,6,8-tetraoxatetrasilocane was purchased from Fisher Scientific. 1-Ethinyl-1-cyclohexanol was purchased from Sigma Aldrich. Elastosil® RT 604 A/B was purchased from Wacker.

### Synthesis of poly(3-cyanopropylmethyl)siloxane(*P*_CN_)

In a 3 L 3-necked round-bottom flask equipped with a magnetic stirrer and a dropping funnel, 3-cyanopropylmethyldichlorosilane (1 kg, 5.49 mol) was added. Mili-Q water (185 mL, 10.28 mol) was added dropwise. Evolving hydrogen chloride was quenched in gas-washing flasks filled with aqueous sodium hydroxide solution. The reaction mixture was stirred overnight at room temperature. The mixture was diluted with dichloromethane. Subsequently, the organic phase was washed with Mili-Q water three times. The organic phase was concentrated using a rotary evaporator under reduced pressure. Finally, the product was dried overnight in a vacuum oven at 100 °C to obtain a slightly yellow oily product. ^1^H NMR (400 MHz, CDCl_3_, *δ*, Fig. S1): 2.36 (m, 2H, CH_2_–CN), 1.65 (m, 2H, C̲H̲_2̲_–CH_2_–CN), 0.67 (m, 2H, Si–CH_2_), 0.11 (m, 3H, Si–CH_3_). ^13^C NMR (400 MHz, CDCl_3_, *δ*, Fig. S2): 119.63 (CN), 20.32 (C̲H̲_2̲_–CN), 19.62 C̲H̲_2̲_–CH_2_–CN), 16.32 (Si–CH_2_), −0.75 (Si–CH_3_). ^29^Si NMR (400 MHz, TMS, *δ*, Fig. S3): −14.62 (Si–OH), −20.31 (–Si(CH_2_)(CH_2_)_3_CN–O–, in cycles), −22.65 (–Si(CH_2_)(CH_2_)_3_CN–O–, in linear chains).

### Ink preparation

Elastosil® RT 604 Part A (21.6 g), *P*_CN_ (16.0 g), and 1-ethinyl-1-cyclohexanol (0.2 g) in a SpeedMixer for ten minutes at 3500 rpm. In the next step, 360 µL Karstedt's catalyst (2.1–2.4% platinum divinyltetramethyldisiloxane in xylene) was added and mixed in the SpeedMixer for 30 seconds at 3500 rpm. Afterwards, 2,4,6,8-tetramethyl-2,4,6,8-tetrakis({3-[(oxiran-2-yl)methoxy]propyl})-1,3,5,7,2,4,6,8-tetraoxatetrasilocane (1.6 g, 2.30 mmol) was added and mixed in the SpeedMixer for 30 seconds at 3500 rpm. Lastly, Elastosil® RT 604 Part B (2.4 g) was added and mixed in the SpeedMixer for 30 seconds at 3500 rpm. The ink was loaded in two 30 mL syringes and degassed in high vacuum for 30 min. If the ink was not used directly, it was stored in the freezer and allowed to warm to room temperature before use.

### Fabrication

The ink was dispensed onto a stainless steel surface treated with Neukadur separating spray. The dielectric film was blade-coated, followed by cross-linking at 100 °C. Thereafter, the electrode was deposited onto the dielectric film using a shadow mask. These two steps were repeated until the desired number of layers was reached, which was 20 active layers in our case.

### Post-processing

After fabricating the stack actuators, they were detached from the stainless-steel substrate and post-cured in an oven at 150 °C overnight to ensure complete cross-linking of the *P*_CN_. To minimize deformation, the films were hung in the oven. Afterwards, the stacks were cut with a razor blade, and the electrodes on the sides were connected using carbon black powder.

## Results and discussion

### Synthesis and characterization of the dielectric ink

**Scheme 1:**
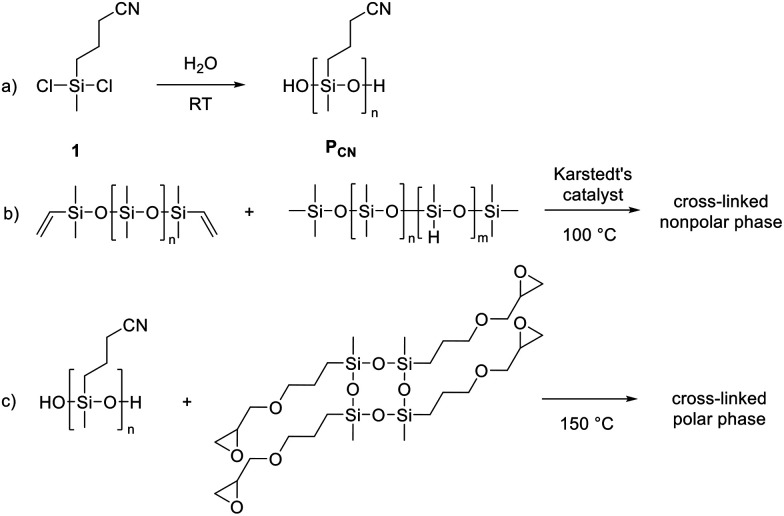
Hydrolysis-condensation of 3-cyanopropylmethyldichlorosilane (1) in the presence of water yields *P*_CN_, which serves as the polar phase in the capillary ink (a). Elastosil® RT 604, which consists of a vinyl end-functionalized PDMS and a poly(dimethylsiloxane-*co*-methylhydrosiloxane) cross-linker, is employed as the nonpolar phase (b). The polar phase is selectively cross-linked at elevated temperature using a commercially available epoxy cross-linker (c).

A common strategy to increase the dielectric permittivity of polysiloxane elastomers is grafting polar side groups onto the polymer backbone.^43^ The nitrile group is particularly promising due to its small volume and strong dipole moment. This, combined with the highly flexible polysiloxane backbone, enables the creation of polar polymers with a low *T*_g_, a key characteristic for achieving polar elastomers. Because polar groups are mobile, they can be easily polarized by an external electric field, thereby increasing the dielectric permittivity *via* dipolar polarization. We synthesized poly(3-cyanopropylmethylsiloxane) (*P*_CN_) *via* a hydrolysis-condensation reaction of 3-cyanopropylmethyldichlorosilane (1) in the presence of water (Scheme 1a). The reaction was successfully scaled up to 1 kg, affording *P*_CN_ in 90% yield, which underscores its potential for commercial production. The product contains approximately 10% cyclic by-product, which could be effectively removed by toluene extraction.^71^

To utilize a high *ε*_r_ polymer as a dielectric in actuators, the material must not only meet all requirements for actuator operation but also satisfy specific processing parameters. First, the formulation, or ink, should be solvent-free processable to avoid solvent interference with underlying layers and the formation of defects. Secondly, its viscosity should be suitable for forming thin, defect-free films on industrially relevant substrates. Additionally, the ink should exhibit a sufficiently long pot life to ensure that the cross-linking reaction does not interfere with the thin-film processing. Premature cross-linking may result in gel particle formation and defects within the thin films. Finally, once processed into thin films, cross-linking should occur rapidly – ideally within seconds – to achieve a fast, efficient process.^72^

**Fig. 1:**
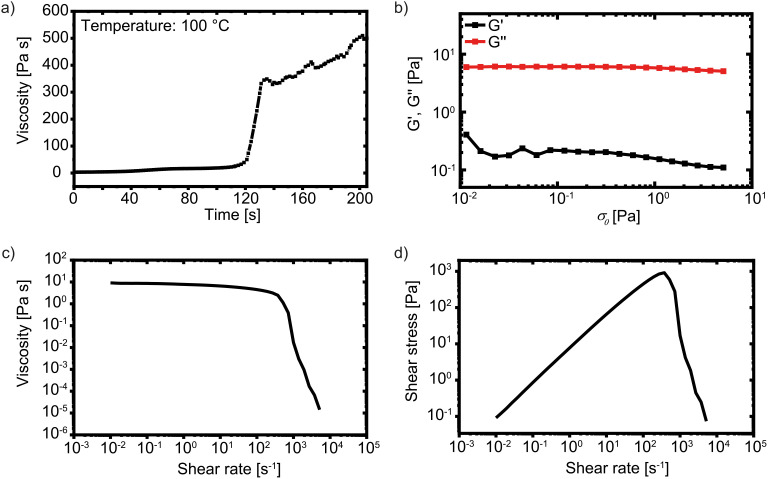
Rheological properties of the capillary ink. At a constant shear of 0.1 s^−1^ at 100 °C, a steep increase in viscosity at 120 s is observed, which indicates the onset of cross-linking (a). Stress-controlled amplitude sweep at 10 rad s^−1^ (b). Shear rate sweep shows viscosity drop at high shear rates (c). Shear rate sweep shows shear thinning behaviour at high shear (d).

One of the most important properties of the high *ε*_r_ ink is that it can be processed into defect-free thin films on common substrates. The quality of the first layer affects the quality of subsequent layers and the performance of the entire stack. However, the as-synthesized *P*_CN_ exhibits pronounced dewetting on industrially relevant substrates, such as stainless steel and glass. In contrast, commercial PDMS formulations can be easily processed to defect-free thin films on steel. We overcome the dewetting problem by using a capillary ink synthesized by mixing *P*_CN_ and an epoxycyclosiloxane cross-linker with a commercial PDMS formulation, Elastosil® RT 604 (Scheme 1).^73^ The polar phase content was kept at 40 wt% to prevent dewetting of the first dielectric layer from the substrate, which occurs if the polar phase content is increased. Owing to the inherent immiscibility of the two components with different polarity, mixing at 3500 rpm yields a stable capillary ink in which the *P*_CN_ dispersed phase is stabilized by the silica particles present in Elastosil®. To enable fast processing of a stack actuator, the cross-linking reaction should occur rapidly at an elevated temperature. Thus, suitable cross-linkers and cross-linking reactions needed to be identified so that the ink could be applied to a hot substrate at 100 °C, remain sufficiently stable at this temperature to allow processing into thin films, and then cross-link within a few minutes to minimize the time required to form a stack. After processing the ink into thin films, the PDMS phase cross-links first, while the polar phase cross-links later during post-curing after stack manufacturing. The Elastosil® formulation consists of PDMS functionalized with vinyl end groups (Scheme 1b) and a poly(dimethylsiloxane-*co*-methylhydrosiloxane) cross-linker. The hydrosilylation reaction between a hydrosilyl and a vinyl group, catalyzed by Karstedt's catalyst, leads to cross-linking. To optimize both the pot life and the cross-linking rate, we carefully adjusted the amounts of Karstedt's catalyst and the thermolabile inhibitor 1-ethinyl-1-cyclohexanol. This reduced the curing time to 2 minutes at 100 °C (Fig. 1a) and enabled fast processing compatible with a layer-by-layer technique, thereby producing stack actuators with up to 20 active layers in just 2 hours. The resulting films exhibit sufficient mechanical stability for easy handling, despite the polar *P*_CN_ phase remaining only partially cross-linked.

The composition of the polar phase was also carefully selected, as the components used should not interfere with the rather sensitive hydrosilylation catalyst, which is easily poisoned. The synthesized *P*_CN_, which contains silanol end groups, can be cross-linked with a commercially available tetrakisepoxy cyclosiloxane at 150 °C (Scheme 1c), eliminating the need for additional end-group functionalization of *P*_CN_. At this elevated temperature, the silanol groups can react with the epoxy moieties *via* ring opening, resulting in effective cross-linking within the polar phase. An additional advantage of using tetrakisepoxy cyclosiloxane is its immiscibility with PDMS, which prevents it from entering the PDMS phase. This phase-selective localization enables epoxy-based cross-linking of the polar domains without interfering with the platinum-catalized curing of Elastosil®. In contrast, other cross-linking strategies, such as thiol–ene chemistry, are incompatible with platinum catalysts, as thiol groups readily coordinate to platinum, rendering the catalyst inactive. Similar incompatibilities arise with tin-catalyzed condensation reactions and with ring-opening polymerizations initiated by strong bases. Finally, the cross-linking of the *P*_CN_ domains is achieved in a post-curing step by heating the layered films at 150 °C overnight in an oven.

The rheological properties of the capillary ink are determined by different rheological tests (Fig. 1). The ink behaves like a Newtonian fluid, with *G*″ consistently exceeding *G*′ as shown by the stress-controlled amplitude sweep (Fig. 1b). A shear rate sweep shows significant shear dependence of viscosity and shear stress (Fig. 1c and d). Up to a shear rate of 400 s^−1^, the viscosity remains constant at 100 Pa s, while the shear stress increases linearly up to 1000 Pa. Above a shear rate of 400 s^−1^, the viscosity and the shear stress decrease rapidly.

### Material properties

The network structure as well as the thermal, mechanical, and dielectric properties of the resulting elastomer were investigated. To verify the chemical cross-linking reactions, the FTIR spectra of the ink and the resulting elastomer were compared (Fig. S4). The completion of the platinum-catalyzed hydrosilylation is confirmed by the disappearance of the vinyl C=C stretching vibration at 1670 cm^−1^ and the appearance of new signals in the 1350–1450 cm^−1^ region, which correspond to the CH_2_ deformation modes of the newly formed ethylene cross-links. Simultaneously, the disappearance of the oxirane ring vibrations at 840 cm^−1^ validates the epoxy-silanol condensation. The broadening of the siloxane backbone signals (1000–1100 cm^−1^) further indicates the formation of Si–O–C ether linkages. These results confirm that the polar phase is selectively cross-linked without hindering the curing of the nonpolar PDMS matrix.

**Fig. 2:**
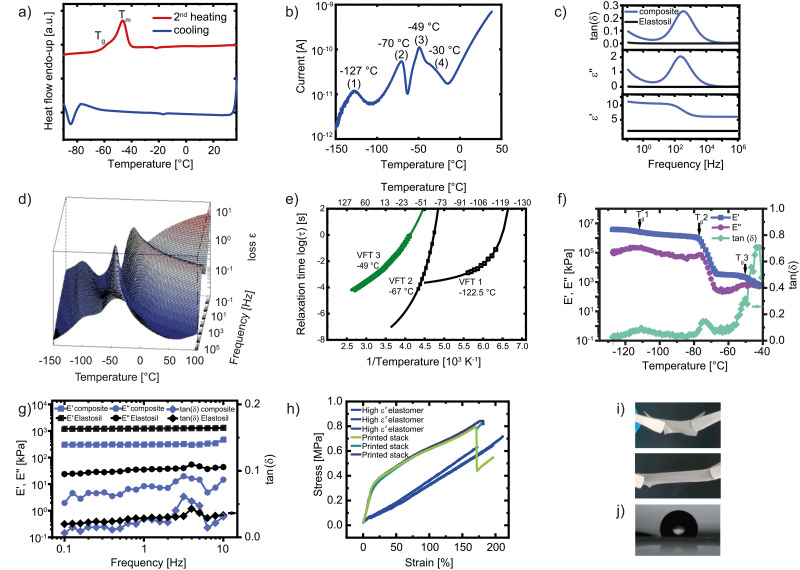
The cooling and second heating curves from DSC measurements conducted at a heating rate of 20 K min^−1^ from −100 °C to 40 °C show a crystallization peak below −80 °C and two overlapping transition temperatures (a). TSDC measurement where the sample was cooled to −150 °C under a small voltage and then heated to 100 °C while recording the depolarization current (b). Dielectric spectroscopy from 0.1 to 10^6^ Hz at room temperature. Our elastomer exhibits a dielectric permittivity of 5.7 at high frequencies and 11.0 at 10 Hz, which is substantially higher than that of Elastosil® (c). 3D plot of conduction-free dielectric loss 
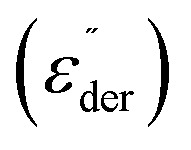
 of the polar elastomer (d) and the Arrhenius relaxation plot with open symbols for VFT fit 1 and closed symbols for VFT fit 2 (e). Temperature-dependent DMA at 1 Hz recorded by cooling the sample from room temperature to −127 °C. Three distinct transitions can be observed (f). Frequency sweep (0.1–10 Hz) at room temperature for our elastomer and pure Elastosil®. Our elastomer exhibits a lower storage modulus (350 kPa) than Elastosil® (1000 kPa), while both materials show similar loss moduli (g). Tensile test of our dielectric elastomer and of the printed stack with eight active layers (h). Photo of the elastomer in the relaxed and stretched state (i). Wetting angle of a water drop on the cross-linked elastomer (j).

Thermogravimetric analysis (TGA) shows the material's thermal stability up to 250 °C (Fig. S5). Decomposition occurs in two steps. The first, at 400 °C, is due to the polar phase, while the second is due to the PDMS phase. About 14% of the residue is due to silica present in the PDMS phase. Fig. 2a shows the second heating curve from a differential scanning calorimetry (DSC) measurement, which shows a *T*_g_ at −59 °C, assigned to the polar phase and overlapping with the PDMS chain melting at −47 °C. The *T*_g_ of the PDMS phase, which occurs below −120 °C, could not be measured by our DSC equipment. However, thermally stimulated depolarization currents (TSDC) is a more sensitive technique in which the material is poled under an electric field at a particular temperature and cooled with the field to sufficiently low temperatures below the sample's *T*_g_, before heating to record the depolarization current. The TSDC curve shows several transitions (Fig. 2b). The first transition at −127 °C is assigned to the *T*_g_ of PDMS, the transition temperature at −71 °C to the *T*_g_ of the bulk polar phase, and the transition at −49 °C is tentatively assigned to the *T*_g_ of the polar *P*_CN_ phase present at the interface with silica particles, resulting in a new interface. The small shoulder at −30 °C is attributed to the melting of the crystalline PDMS domains and is supported by the literature.^74,75^

The frequency-dependent dielectric permittivity at room temperature exhibits two distinct plateau regions (Fig. 2c), which we assigned to the different relaxation times of the nitrile groups in the polar *P*_CN_ phase, either in the bulk or at the interface with the silica particles. At high frequencies (10^6^ Hz), the relative permittivity is 5.7, which increases to 11 at 10 Hz. At high frequencies, only the nitrile groups present in the bulk *P*_CN_ have sufficient mobility to respond. In contrast, the nitrile group on the *P*_CN_ present at the interface with silica exhibits restricted mobility and thus a delayed response in impedance spectroscopy. It can therefore only contribute to permittivity at lower frequencies.^70^ This relaxation has a maximum in tan *δ* at 316 Hz. These interfaces are also affected by moisture, as evidenced by a shift in their relaxation peak to a lower frequency after annealing the samples at 120 °C. Although the annealing procedure does not completely remove water molecules, it shifts the relaxation of the nitrile groups at the interfaces to lower frequencies (Fig. S6). Despite this frequency dependence, the material remains highly stable in the MHz range and maintains a dielectric permittivity significantly higher than that of PDMS across the entire measured spectrum (*e.g.*, Elastosil® ∼ 2.9). This ensures that the *P*_CN_-based material provides superior performance for dielectric elastomer actuators compared to PDMS, even in high-frequency applications where interfacial polarization is diminished.

To better understand the dielectric behavior of the elastomer, dielectric properties were measured as a function of temperature from −150 to +100 °C with a 2.5 K step increase. The 3D loss plot of the sample as a function of both frequency and temperature is shown in Fig. 2d. We observe three relaxation processes whose peaks shift to higher temperatures with increasing frequency. Fitting these relaxation loss peaks to the Havriliak–Negami equation results in an Arrhenius plot (Fig. 2e) with all three processes exhibiting Vogel–Fulcher–Tammann (VFT) behavior, characteristic of glass transitions. At a relaxation time of 100 s (log *τ* = 2 s), corresponding to the dynamic glass transition temperature of a particular relaxation process, we obtain values of −122.5 °C, −67 °C, and −49 °C that match well with the respective TSDC peaks. For the ‘VFT 3’ process that corresponds to the interfacial *T*_g_ relaxation, we obtain high HN fitting shape parameters of *α*_HN_ = 0.84 and *β*_HN_ = 0.91, pointing to water-influenced interfacial regions as observed previously in sulfonyl-modified homo- and co-polymers of polysiloxanes.^45^ This further exemplifies the role of water molecules in the relaxations of polysiloxane-based polymers. The absence of a relaxation process expected from the melting of PDMS crystals may be due to the possible overlap of the process with the bulk *T*_g_ relaxation that happens in this temperature range. However, by employing the temperature derivative of permittivity (*α*_*ε*_) introduced by Wübbenhorst *et al.*,^76,77^ which can reveal hidden processes, we identify a frequency-independent peak maximum at −35 °C that can be correlated with a melting transition (Fig. S7).

Temperature-dependent dynamic mechanical analysis (DMA) of the material, recorded during cooling from room temperature to −128 °C, revealed three distinct thermal transitions (Fig. 2f). The first transition at −110.8 °C corresponds to the *T*_g_ of the nonpolar PDMS phase. The second transition, observed at −74.8 °C, is attributed to the crystallization of the PDMS crystalline domains, while the third transition at −50.2 °C corresponds to the *T*_g_ of the polar *P*_CN_ phase.

We further investigated the mechanical properties of the composite using DMA with a frequency sweep at room temperature (Fig. 2g). Our elastomer exhibited a lower storage modulus (350 kPa) than Elastosil® (1000 kPa), indicating increased softness. For both materials, the storage modulus was frequency-independent, indicating a predominantly elastic response. The loss modulus of the elastomer was slightly lower than that of Elastosil®, and the tan *δ* remained below 0.1 across all frequencies, further confirming the excellent mechanical properties of our dielectric elastomer. Tensile test (Fig. 2h) yielded a Young's modulus of 330 kPa. The elastomer can be easily stretched by hand and returns to its initial shape (Fig. 2i). The strain at break of 175% is sufficiently high for actuator applications. Additionally, a staked actuator sample consisting of 8 capacitive layers stacked on top of one another was subjected to tensile testing (Fig. 2h). The Young's modulus of this sample was 1600 kPa, thus significantly higher than that of the neat dielectric elastomer. A closer look at the stress–strain curve shows a large Young's modulus at small strains. Above a strain of 15% a flattening of the curve is observed. The strain at break of these printed actuators is 170%, very similar to that of the neat dielectric elastomer. Thus, it can be concluded that the electrode material has a great influence on the actuator's mechanical properties, especially at low strains. A higher Young's modulus of the applied electrode may result in reduced actuation strain in the stacks, as the electrodes act as a mechanical constraint that resists. This constraint is most significant at low deformations, where the stiffness mismatch between the electrode and the elastomer is highest. While this reduces the achievable strain, the current electrode configuration ensures excellent reliability and robustness for high-voltage operation. Future device-level optimization will focus on reducing the electrode thickness to mitigate this mechanical constraint while maintaining high electrical conductivity. Cyclic tensile testing shows only a small hysteresis for both the dielectric elastomer and the printed stack, further confirming the excellent mechanical properties (Fig. S8).

Despite the rather high polar-phase content, the surface of our film is hydrophobic, exhibiting a wetting angle of 122° (Fig. 2j). This behavior is expected because the polar *P*_CN_ phase is embedded within the hydrophobic non-polar PDMS matrix and will impact the printability of the electrode ink.

### Single-layer actuator

**Fig. 3:**
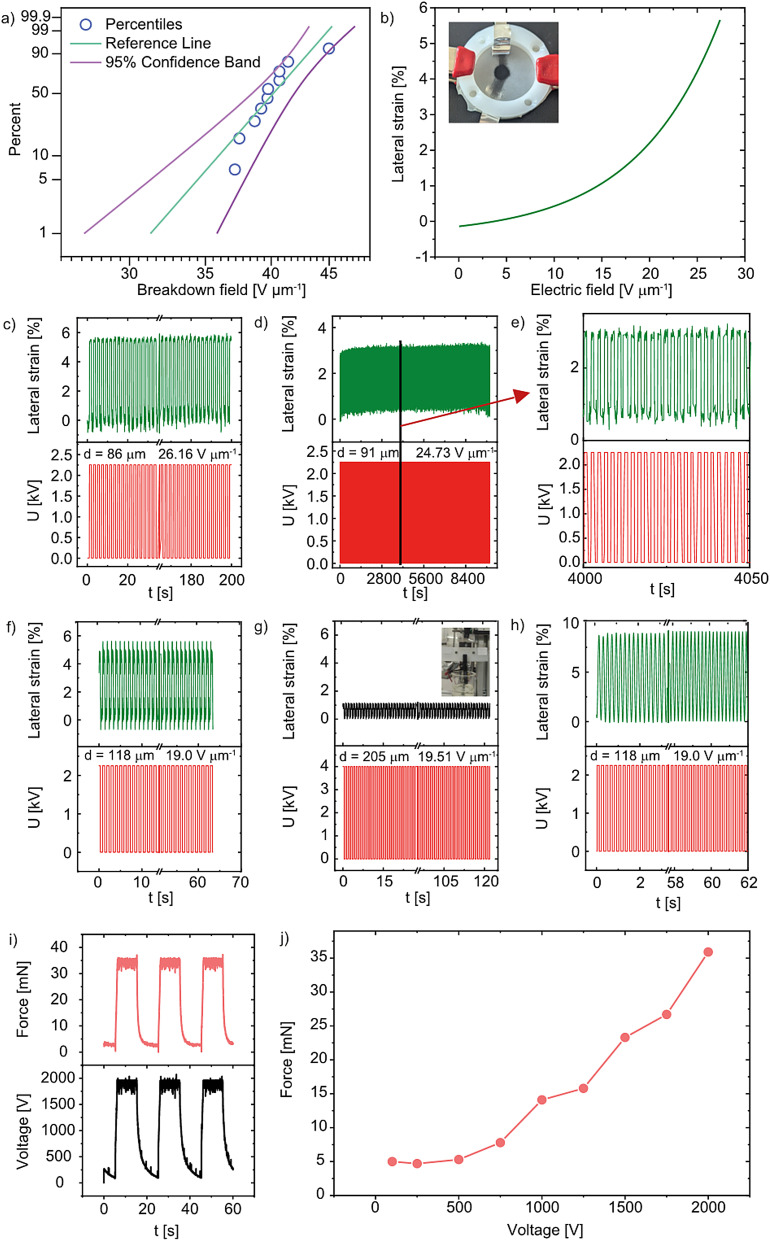
Weibull plot of dielectric breakdown field. Most of the samples have a breakdown field of below 41 V µm^−1^ (a). Stepwise increases in the electric field lead to progressively larger actuation strains in circular actuators (b). Cyclic actuation performance of single-layer actuators at 26.2 V µm^−1^ yields a maximum strain of 5.7%, which remains stable over 100 cycles at 1 Hz (c). The insert shows the photo of the circular actuator. The actuator survived more than 5000 cycles at 1 Hz and 24.7 V µm^−1^ (d) with the actuation between 4000 and 4050 s enlarged (e). Cyclic actuation of stripe actuators constructed from our elastomer tested at 1 Hz, 19.0 V µm^−1^, exhibiting 5.5% lateral actuation strain (f); of Elastosil at 1Hz 19.5 V µm^−1^ exhibiting only 1.1% lateral strain (g), and of our elastomer under resonance conditions at 5 Hz and 19.0 V µm^−1^ exhibiting a 9% lateral actuation strain for over 300 cycles (h). Force of actuation of stripe actuator at 2000 V (16.9 V µm^−1^). The stripe is prestrainded with 0.2 N. The measured force is 35.9 mN (i). The force of a stripe actuator increases gradually with voltage (j).

First, we determined the breakdown field of 100 µm-thick elastomer film by sandwiching it between two rigid electrodes with a 1 mm^2^ area and applying increasing voltages. The results are shown in a Weibull plot (Fig. 3a), which exhibits a scale parameter of 41 and a shape parameter of 17. All samples withstood an electric field of 35 V µm^−1^. Most dielectric breakdowns occurred around 41 V µm^−1^, and above 45 V µm^−1^, almost all samples failed.

We then evaluated the performance of the synthesized dielectric elastomer in single-layer circular actuators. An about 100 µm-thick elastomer film was cut into circular specimens, biaxially prestrained by 5%, and mounted between two rigid plastic frames with an inner diameter of 25 mm. Carbon black electrodes with a diameter of 6 mm were then brushed in the middle on both sides of the elastomer. A high-voltage source is connected to the actuator *via* aluminium strips. A camera detects the edge of the carbon black electrode, and the change in the diameter is recorded. The voltage applied to the actuator was increased in 100 V steps up to the breakdown voltage, with each voltage held for 2 seconds before the next increase. Up to an electric field of 14 V µm^−1^, only a slight increase in actuation was observed. Above this field, the actuation increased steeply to a maximum of 5% before reaching a breakdown voltage of 27.4 V µm^−1^ (Fig. 3b).

We also evaluated the performance of the circular single-layer actuators under varying electric fields at 0.1 Hz. Already at a rather low electric field of 8.7 V µm^−1^ (750 V), the actuator exhibits 1.25% lateral actuation stable over the 10 recorded consecutive cycles (Fig. S9), indicating negligible viscous losses of our material. The actuation strain increases with electric field strength, and at 20.4 V µm^−1^, the lateral strain reaches 4.8% (Fig. S10–S13). A further increase in the field to 26.2 V µm^−1^ increases the actuation strain to 5.7% (Fig. 3c), which exhibited no hysteresis over the 100 cycles investigated at 1 Hz. The actuation was the same at 0.1 Hz and 1 Hz (Fig. S14–S15), indicating frequency-independent performance within this range. The actuator was also stable over 5000 cycles at 1 Hz at an electric field of 24.7 V µm^−1^ (Fig. 3d).

We also tested our material in a stripe actuator, which was constructed by clamping the dielectric film (3 cm × 1 cm and 118 µm thick) coated with two carbon-black electrodes on each side, leaving about 2 mm of uncoated dielectric on both sides to prevent electrical breakdown (Insert Fig. 3g). The actuator was positioned between two rectangular frames, one at the top and one at the bottom. A plastic disc was attached to the bottom frame to slightly prestrain the actuator and prevent damping by suspending the disc in an oil bath. During actuation, the bottom frame, which clamps the actuator, moved up and down, and this motion was recorded by a laser to provide lateral actuation.^8^ At an electric field of 19 V µm^−1^ and a frequency of 1 Hz, our material exhibits a lateral actuation strain of 5.5% (Fig. 3f), which is five times higher than that of the Elastosil stripe actuator (1.1%, Fig. 3g). When the frequency is increased to 5 Hz, the actuator constructed from our material enters the resonance region, achieving an over 9% lateral actuation strain at the same electric field (Fig. 3h) while maintaining stable performance for more than 300 cycles. Additionally, we measured the force of the stripe actuator. For that, we use a 2 N load cell and apply a prestrain of 0.2 N to the stripe actuator. At an electric field of 16.9 V µm^−1^ the stripe actuator exhibits 35.9 mN (Fig. 3i) for an actuator stripe weighing 0.13 g. The force increases gradually with increasing electric field (Fig. 3j).

### Stack actuator

The force generated by single-layer actuators is rather small. By placing many single-layer actuators on top of one another, a stack is formed for which the actuation in thickness as well as the force generated increases with the number of layers.^78,79^ Such stacks can be fabricated in two ways: either by using preformed films or by a “wet” layer-by-layer method. The first approach uses thin elastomer films coated with electrodes, similar to single-layer actuators, and places them on top of one another to form a stack actuator.^79–82^ However, this method often leads to poor interlayer adhesion and incomplete bonding, causing performance losses. The second method employs a layer-by-layer fabrication process in which dielectric and electrode layers are deposited in alternating fashion, eliminating the need for manual stacking.^16,83–85^ Often, the dielectric layers are blade-cast into thin films, which are cross-linked, followed by electrode patterning *via* screen printing or spraying. This cycle is repeated several times to build the multilayer structure.

**Fig. 4:**
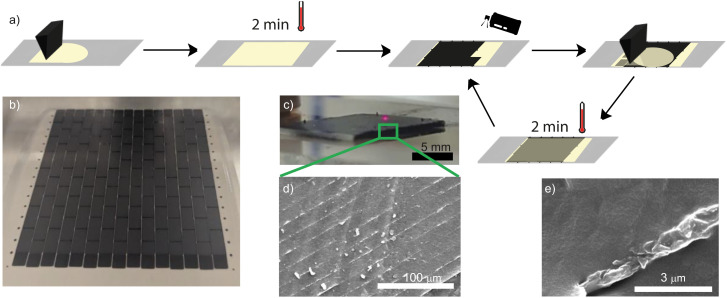
Scheme of the layer-by-layer fabrication process of the stack actuator. Dielectric polymer layers are blade-cast and cross-linked at 100 °C within 2 min. Electrodes are sprayed onto each dielectric layer and dried before the subsequent layer is applied (a). The final polymer sheet, consisting of 225 stacked actuators, was dried in an oven at 150 °C overnight (b). Photo of a stack actuator consisting of 20 active layers (c). SEM cross-sectional image of the stack actuator comprising 20 active layers, each dielectric having a thickness of 20 µm, and thin and uniform layers of electrodes with a thickness below 1 µm. The layers' thicknesses are highly uniform and homogeneous (d). High-magnification SEM image of a single electrode layer, revealing a smooth surface (e).

Here, we use a layer-by-layer approach to manufacture stack actuators, in which the dielectric layer is blade-cast onto a preheated metal substrate and thermally cured for 2 minutes (Fig. 4a). We then sprayed the electrode ink through a shadow mask onto the dielectric. This process is repeated as many times as necessary until the stack actuator reaches the desired number of layers. The printed multilayer structure was allowed to cool, detached from the metal substrate, and post-cured overnight at 150 °C to complete cross-linking of the polar phase (Fig. 4b). Finally, we cut individual actuators from the multilayer film using a razor blade, yielding 225 stack actuators, each comprising 20 active layers (Fig. 4c). The quality of the multilayer structure was investigated using scanning electron microscopy (SEM) on the cross-section. The SEM image (Fig. 4d) reveals uniform dielectric layers (dark) alternating with bright electrode layers. All dielectric layers are 20 µm thick, and the thickness is constant over all layers. A closer view of a single electrode layer (Fig. 4e) reveals a thickness of approximately 1 µm. The electrode's smooth surface is crucial, as any inhomogeneities can lead to hot spots and dielectric breakdown.

**Fig. 5:**
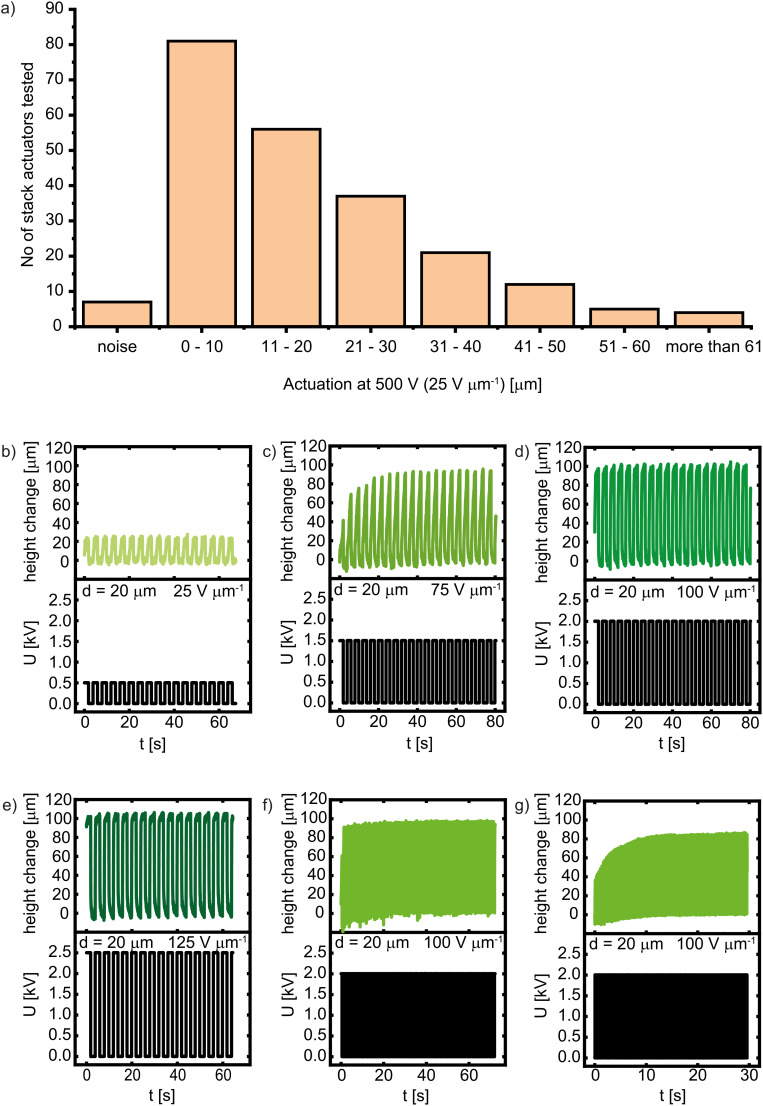
Stack actuator performance at different electric fields and frequencies. The stack actuators consist of 20 active layers, each 20 µm thick. Histogram performance of 225 modules at 500 V, an electric field of 25 V µm^−1^, and 100 mHz. Only 7 modules showed no actuation. The biggest group with 81 stack actuators showed an actuation strain between 0–10 µm, 4 actuators achieved an actuation strain above 61 µm (a). An actuator tested at 500 V (25 V µm^−1^) and 0.25 Hz reached hight change of 25 µm (b), at 1500 V (75 V µm^−1^) and 0.25 Hz, the hight change increases to 93 µm after an initial build-up over four cycles (c), at 2000 V (100 V µm^−1^) and 0.25 Hz, the hight change increases to 100 µm (d), at 2500 V (125 V µm^−1^) and 0.25 Hz, the actuator immediately reached a hight change of 105 µm (e), at 2000 V (100 V µm^−1^) and 1 Hz, the actuator maintains a hight change of 97 µm (f), at 2000 V (100 V µm^−1^) and 10 Hz, the actuator shows initial build-up before stabilizing at 85 µm height change (g).

All stack actuators were tested at 100 mHz and 500 V, corresponding to an electric field of 25 V µm^−1^, while recording the height change during actuation using a laser. Their actuation performance is presented in Fig. 5a. A number of 225 stack actuators were tested, of which only 7 (3%) actuators showed no actuation (Fig. S16), 81 (36%) actuators showed a height change between 1 and 10 µm (Fig. S17), 4 (2%) actuators showed a height change of more than 61 µm (Fig. S18–S23). The observed performance distribution is primarily caused by elastomer defects and mechanical damage incurred during the sample-cutting process. Such defects can introduce micro-cracks or lead to premature electrical breakdown, thereby limiting the actuation strain in the affected samples. In contrast, electrode deposition uniformity may also influence this variation. Future studies will aim to mitigate these inconsistencies by transitioning to high-precision cutting methods to minimize cutting defects. Additionally, we evaluated the performance of individual stack actuators under varying electric fields. At 500 V, corresponding to 25 V µm^−1^, the actuator reached a height change of 25 µm (6.25%) (Fig. 5b). This actuation at low electric fields significantly outperforms many reported silicone-based stack actuators, which typically require much higher fields to achieve comparable strains (Table S1). This performance remained stable over 17 cycles without any observable degradation. Increasing the voltage to 1000 V, corresponding to 50 V µm^−1^, resulted in a small increase in height change to 26 µm (Fig. S24). A further increase in voltage to 1500 V, corresponding to an electric field of 75 V µm^−1^, increased the height change to 93 µm (Fig. 5c), with a gradual build-up over the first four cycles before stabilizing. Further increasing the electric field to 2000 V (100 V µm^−1^) and 2500 V (125 V µm^−1^) did not significantly enhance the height change, likely because mechanical constraints imposed by the passive regions surrounding the active area limit actuation, or the electrode material is not conducting at these strains (Fig. 5d and e). The actuator was also tested at 100 V µm^−1^ and at 0.25, 1, and 10 Hz (Fig. 5f and g). A small decrease in actuation with the frequency was observed. At 10 Hz, the actuation shows an initial build-up, then stabilizes and remains consistent at 85 µm height change across all cycles.

The long-term reliability of the stack was validated through an extended endurance test. A stack module operated continuously at 0.25 Hz showed no loss in actuation performance over more than 18.3 h, successfully completing more than 16 500 cycles (Fig. S25). This confirms the material's high fatigue resistance and the stability of the multilayer architecture under repeated actuation.

Owing to its robust and scalable synthesis, solvent-free thin-film processability, long pot life, rapid on-demand cross-linking, and excellent mechanical and dielectric properties enabling low-voltage actuation, our material is a strong candidate for soft robotic applications.

## Conclusions

In this work, we developed a capillary-processable ink that uniquely combines a long pot life, the ability to form defect-free thin films on hot substrates, and rapid cross-linking within two minutes. This unprecedented combination of processing characteristics enabled precise, reproducible, and scalable fabrication of multilayer stack actuators. The ink's extended working time ensures consistent deposition, while its fast curing kinetics align with industrial manufacturing requirements and support rapid layer buildup without compromising film quality.

Beyond its processing advantages, the cross-linked material exhibits excellent mechanical, dielectric, and electromechanical properties, *e.g.* a storage modulus of 350 kPa, a low mechanical loss below 0.05, and a dielectric permittivity of 11. These material characteristics, together with the ink's robust processing window, provide the foundation for fabricating stack actuators with exceptional structural uniformity and reliable electromechanical performance. The actuators manufactured with this ink exhibit stable electromechanical response, highlighting the synergy among the ink's formulation, processing behavior, and final material functionality. Stack actuators comprising 20 active layers, each 20 µm thick, achieve a 25 µm height change at 500 V, corresponding to 25 V µm^−1^. The actuation remains stable, over 300 cycles at 100 V µm^−1^ 10 Hz with no hysteresis between cycles. A further increase in voltage to 1500 V, corresponding to an electric field of 75 V µm^−1^, increased the height change to 93 µm. The actuation remains stable, over 300 cycles at 10 Hz with no hysteresis between cycles.

Overall, this work presents a previously unavailable combination of processability and material performance for soft actuator manufacturing. The ink platform introduced here constitutes a significant step toward scalable, high-precision production of multilayer actuators. It establishes a foundation for future advances in soft robotics, electromechanical systems, and large-area device manufacturing.

## Author contributions

J.W. and P.M.D. synthesized the polymer. J.W. formulated the ink, prepared the actuators, conducted the rheological tests, DMA measurements, tensile test measurements, and actuator measurements. T.R.V. conducted the dielectric relaxation spectroscopy investigations. V.R.L. conducted the force measurements. J.W., T.R.V., and D.M.O. wrote the original draft. D.M.O. initiated the activity, designed the materials, acquired funding, and coordinated and supervised this research. All authors contributed to discussions, reviewing, and editing, and have approved the final version of the manuscript.

## Conflicts of interest

P. M. D. and D. M. O. have a patent application comprising the capillary suspension of polar and non-polar polysiloxanes. The remaining authors declare no competing financial interests.

## Supplementary Material

MH-013-D6MH00302H-s001

## Data Availability

The data supporting the findings of this study are available within the article and its supplementary information (SI). Supplementary information: ^1^H, ^13^C and ^29^Si NMR spectra, TGA, dielectric spectroscopy data, tensile tests, and actuator tests. See DOI: https://doi.org/10.1039/d6mh00302h. The raw data generated and analyzed during the current study were uploaded to Zenodo: https://doi.org/10.5281/zenodo.18416344.
