# Supplementary material for: Electrically responsive multilayer soft actuators using a solvent-free high dielectric permittivity polysiloxane ink
Source: Mater Horiz. 2026 Apr 1;13(11):5502–14. doi: 10.1039/d6mh00302h (PMC13070309; doi:10.1039/d6mh00302h)
Supplement: MH-013-D6MH00302H-s001 [file MH-013-D6MH00302H-s001.pdf]

Supplementary Information (SI) for Materials Horizons.
This journal is © The Royal Society of Chemistry 2026

Supporting Information

Electrically Responsive Multilayer Soft Actuators Using a Solvent-Free High Dielectric Permittivity Polysiloxane Ink

*Jana Wolf, Patrick M. Danner, Thulasinath Raman Verkatesan, Valentin Razvan Lupu, and Dorina M. Opris**

Rheological characterization: All rheological measurements were performed on a stress-controlled Anton Paar MCR 702 rheometer. Amplitude sweeps and flow curves (shear rate sweeps) were performed using a parallel plate geometry with a 25 mm diameter upper plate and a 25 mm diameter bottom plate connected to a Peltier element set at 25 °C. Amplitude sweeps were performed at an angular frequency of 10 rad s^{-1}, and shear stress was ramped in logarithmically spaced steps from 0.1 to 500 Pa. Flow curves (shear rate sweeps) from 0.01 to 8'630 s^{-1} were conducted in logarithmically spaced steps with durations ranging from 200 s to 10 s to guarantee that samples were in steady state during data acquisition. Curing experiments were performed using parallel-plate measuring geometries. For these measurements, the rheometer was coupled to a CTD 600 (Convection Temperature Device) to ensure homogeneous, precisely controlled heating of the samples. The temperature was set to 100 °C, and the ink was placed onto the hot plate. The viscosity of the sample was measured at a shear rate of 0.1 s^{-1}. The measurement was started immediately, without waiting for thermal equilibrium.

FTIR: IR spectra were recorded in the range of 4000–600 cm^{-1} on a Bruker Tensor 27 FT-IR spectrometer equipped with an ATR setup.

Tensile measurements: Tensile tests and cyclic uniaxial tensile stress tests were performed on a Zwick Z010 machine. Tensile test specimens were dog-bone-shaped with a gauge width of 2 mm and a gauge length of 18 mm. They were prepared by die-cutting. The applied preload force was 0.005 N, and the sample was stretched at 50 mm min^{-1}. The strain was determined using a traverse-mounted sensor. For the cyclic uniaxial tensile stress tests, the samples were strained to 20%, held at this strain for 2 s, and then relaxed. After holding the relaxed position for 2 s, the next stretching cycle started. In total, 20 cycles are measured. At least five samples were measured and averaged. A linear fit to the data determined Young's modulus points over 0-10% strain.

Dynamic mechanical analysis (DMA): Dynamic mechanical analysis was performed on an RSA 3 from TA Instruments. Stripes with a width of 10 mm and a length of 25 mm were measured. The sample was measured at least three times at room temperature for each method to determine average values. For frequency-dependent measurements, a strain of 2% and a preload force of 2 g were applied, and the frequency increased from 0.1 to 10 Hz. For the temperature-dependent measurement, a frequency of 1 Hz and a strain of 0.5% were used. The measurements were conducted in a temperature range from 60 to −127 °C.

Broadband Dielectric Spectroscopy: The dielectric properties were evaluated using a Novocontrol Alpha-A Frequency Analyzer equipped with a Quatro cryosystem temperature control. The dielectric spectra were recorded by applying an external AC voltage of 1 V in the frequency range of 0.01 Hz to 1 MHz at various temperatures. For generating the 3D plot, temperature derivative curve, and fitting the dielectric loss curves, the DCALC program developed by Wübbenhorst was used.[1,2]

Thermally stimulated depolarization current (TSDC): TSDC measurements were conducted using a Keysight electrometer with a built-in DC voltage source. The films were poled at 100 °C using a 5 V μm^{-1} field for 10 min. Thereafter, the temperature was decreased to -150 °C while the voltage was maintained constant. The films were then heated at 5 K min^{-1} using a Novocontrol Quatro cryosystem under a nitrogen atmosphere.

Circular single-layer actuators: Actuation tests were performed with circular membrane actuators. The polymer films were biaxially prestrained by 5% and fixed between two circular plastic frames. Carbon black powder was applied to each side of the film with a brush, forming circular electrodes. The electrodes were connected to a FUG HCL-35-12500 HV power supply with aluminum foil. The actuation strain was determined optically using a digital camera, which detected the edge between the black electrode and the light silicone film, thereby measuring the electrode's diameter extension. The actuators were tested at frequencies between 0.1 and 10 Hz and voltages between 0 and 5.6 kV (the maximum of the voltage source).

Stripe actuators: Actuation tests were performed with stripe actuators. The polymer films were fixed between two rigid frames spaced 1 cm apart. Carbon black powder was applied to each side of the film with a brush. The electrode dimensions are 3 × 1 cm. The electrodes were connected to a FUG HCL-35-12500 HV power supply with aluminum foil. To fix the aluminum foil, the stripe is also clamped between the two rigid frames. The actuation strain was determined with an optoNCDT 2300 laser, measuring the distance from the laser to the bottom frame of the stripe actuator. The actuators were tested at frequencies between 0.1 and 10 Hz and voltages between 0 and 5.6 kV (the maximum of the voltage source).

Force measurements: The stripe actuator was fixed with hooks between a fixed plate and a 2 N load cell. A prestress of 0.2 N was applied. The actuators were tested at voltages between 0.1 and 3 kV.

Stack actuator testing: The stack actuator was placed on a glass slide with sputtered gold electrodes, which were connected to a FUG HCL-35-12500 HV power supply. The actuation strain was determined with an optoNCDT 2300 laser, measuring the distance from the laser to the top of the stack actuator. The actuators were tested at frequencies between 0.1 and 10 Hz and voltages between 0 and 5.6 kV (the maximum of the voltage source).

NMR spectroscopy: ^{1}H, ^{13}C, ^{29}Si, NMR spectra were recorded at 298 K on a Bruker Avance 400 NMR spectrometer using a 5 mm broadband inverse probe at 400.13 and 100.61 MHz. Chemical shifts (δ) in ppm are calibrated to residual solvent peaks (Tetramethylsilane: δ = 0; $CDCl_3$: δ = 7.26 and 77.16 ppm).

Differential scanning calorimetry: DSC investigations were undertaken on a PerkinElmer Pyris Diamond instrument under a nitrogen flow (50 mL min^{-1}) in aluminum crucibles sealed with pierced lids, containing about 10 mg of sample. Two heating and one cooling steps, each with a heating and cooling rate of 20 K min^{-1}, were performed per measurement. The second heating step was used to evaluate the T_g.

Thermalgravimetric analysis (TGA): TGA was performed with a Netzsch TG 209-F1 with a vacuum-tight thermo-microbalance. Samples were heated in an Al_2O_3 crucible at 20 K min^{-1} under airflow.

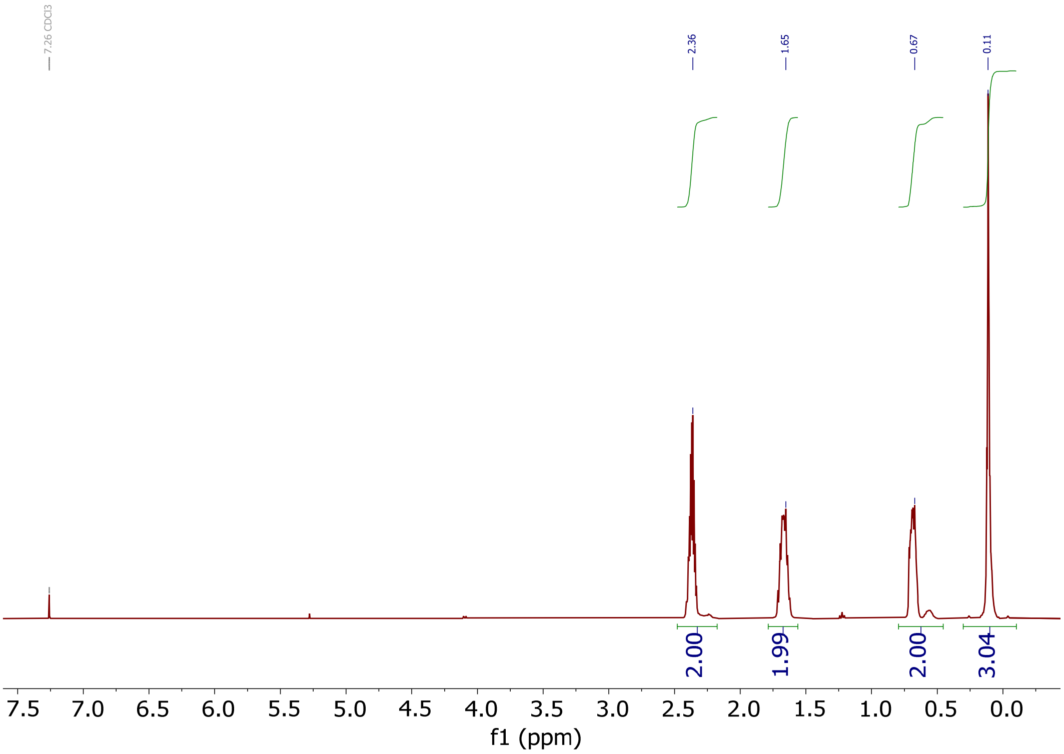

Figure S1. ^{1}H NMR spectrum of $\mathbf{P_{CN}}$ with silanol end-groups. The ^{1}H NMR was recorded in $CDCl_3$.

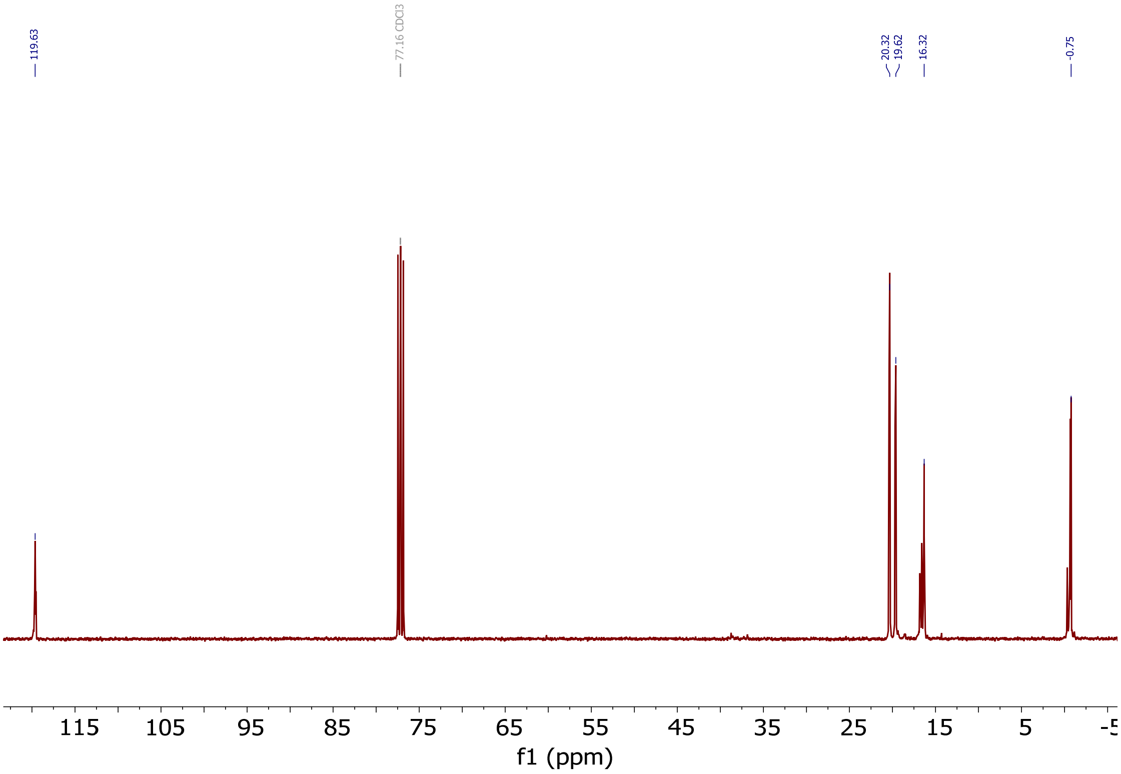

Figure S2. ^{13}C NMR spectrum of $\mathbf{P_{CN}}$ with silanol end-groups. The ^{13}C NMR was recorded in $CDCl_3$.

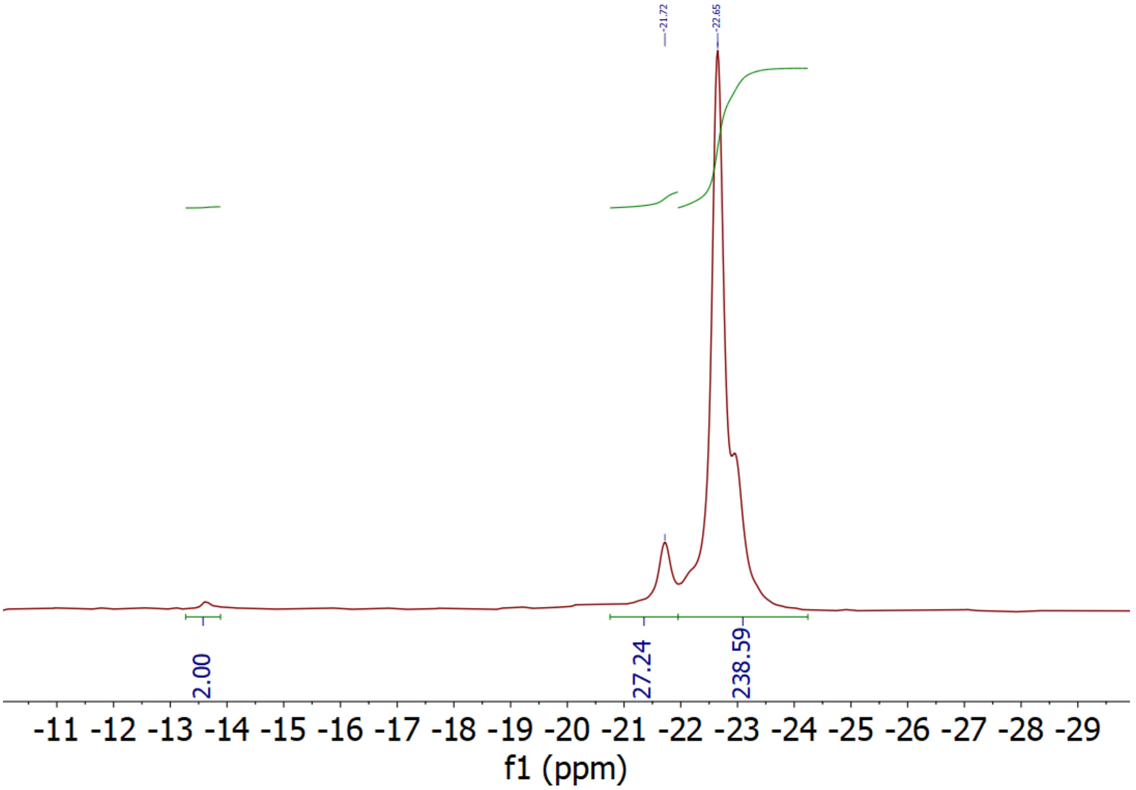

Figure S3. ^{29}Si NMR spectrum of $\mathbf{P_{CN}}$ with silanol end-groups. The ^{29}Si NMR was recorded with $Cr(acac)_3$ as a relaxation agent and TMS as a reference.

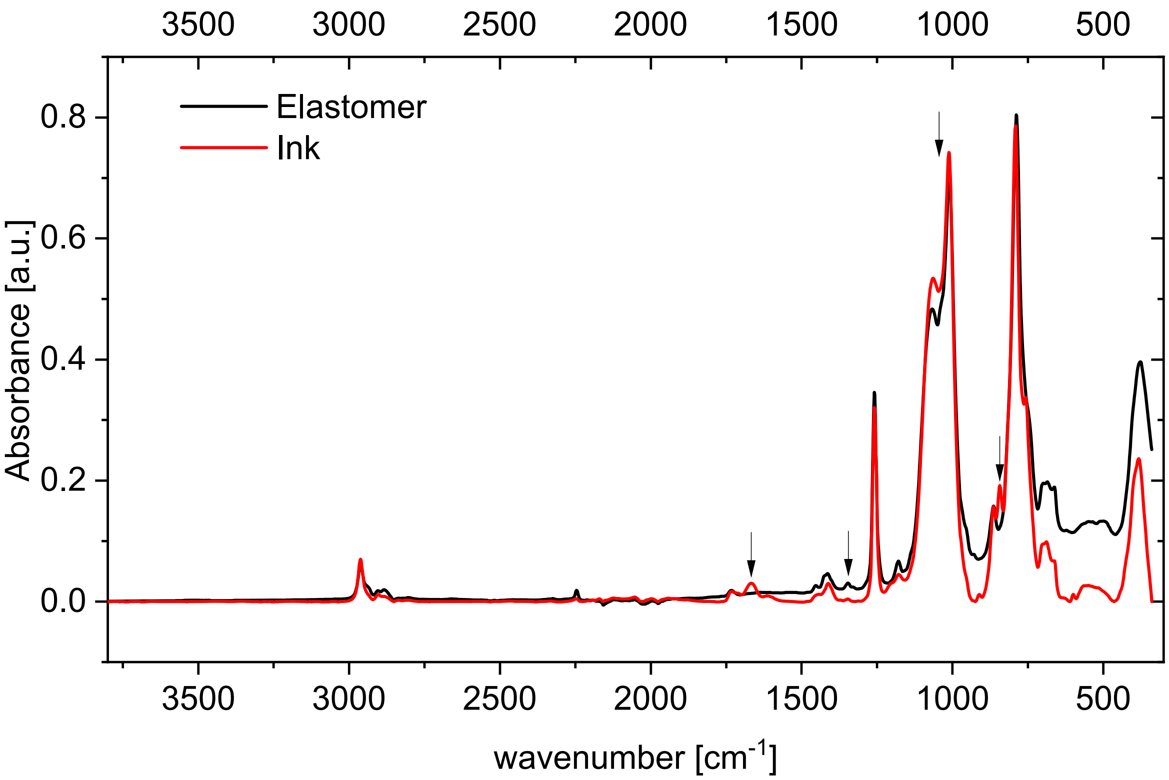

Figure S4. FTIR of polymer ink and corresponding elastomer. The arrows indicate the changes seen before and after cross-linking. The small changes are caused by the low concentration of end-groups present, which are cross-linked.

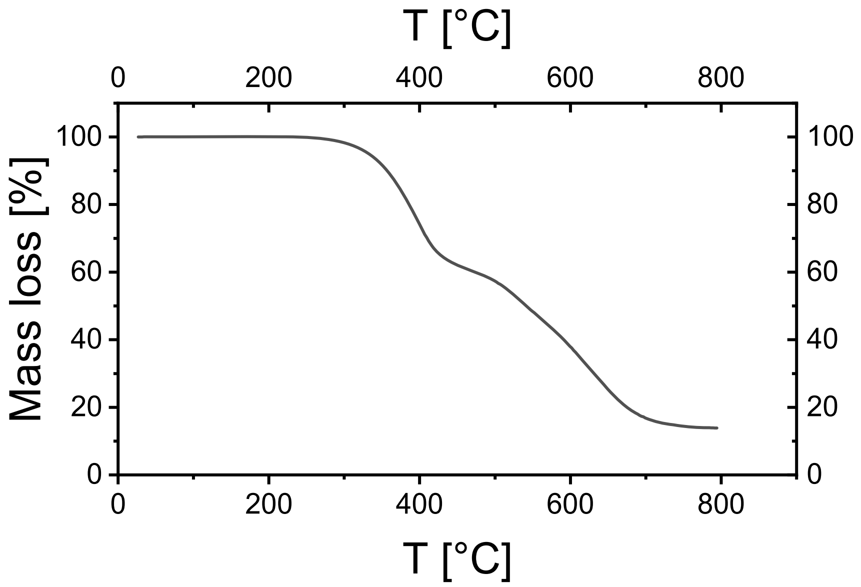

Figure S5. TGA measurement of the composite. The sample was heated in an Al_2O_3 crucible at 20 K min^{-1} under airflow. The material is stable up to 250 °C; a 5% mass loss is observed at 332 °C.

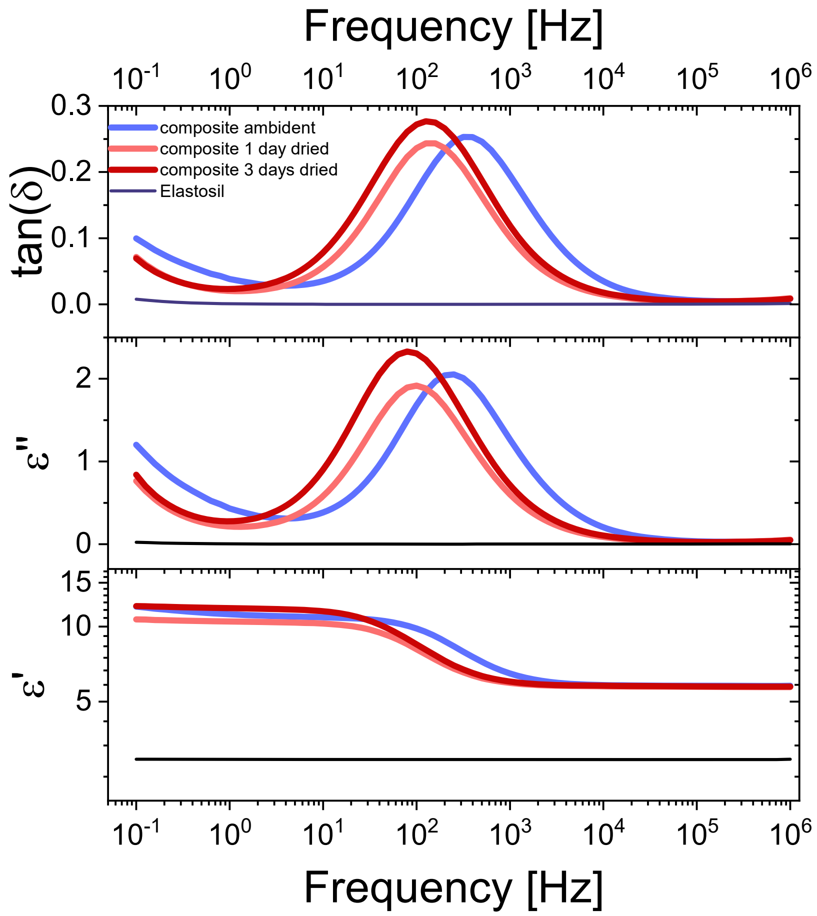

Figure S6. Dielectric spectroscopy from 0.1 to 10^6 Hz at room temperature. Our elastomer exhibits a dielectric permittivity of 5.7 at high frequencies and 11.0 at 10 Hz, which is substantially higher than that of Elastosil®. Drying of the elastomer shifts tan δ to lower frequencies.

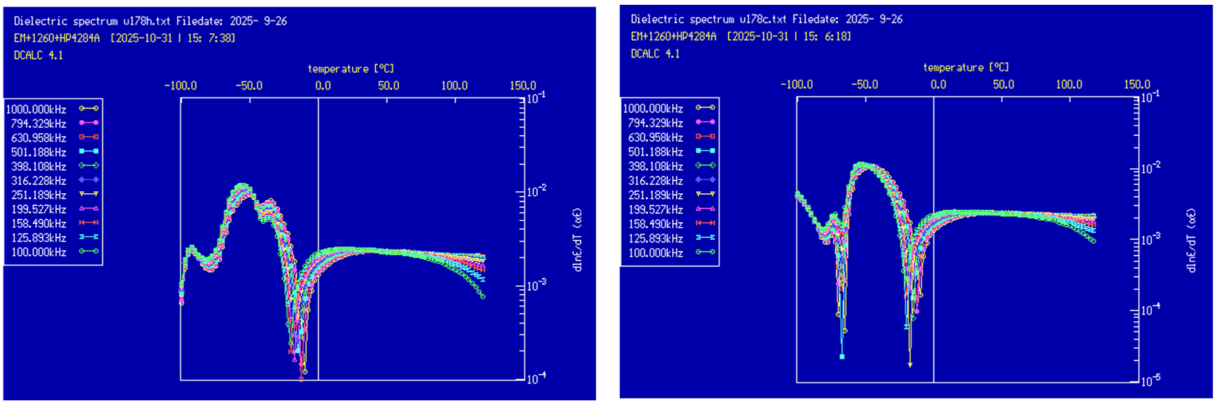

Figure S7. In permittivity temperature coefficient, a type of derivative analysis which is especially useful for phase transitions, we see a frequency-independent peak around -35 °C during heating from -100 °C to 150 °C (Left) and at -75 °C during cooling from 120 °C to -100 °C (right), corresponding to melting and recrystallization, respectively.

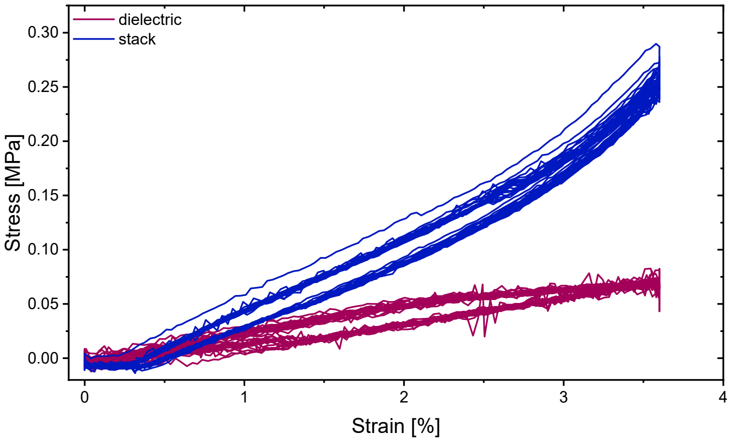

Figure S8. Cyclic tensile test of dielectric elastomer (violet) and stack with electrode (blue). The sample is stretched to 3.6 % strain, held for 2 s, and then relaxed. After holding for another 2 s, the test is repeated 20 times.

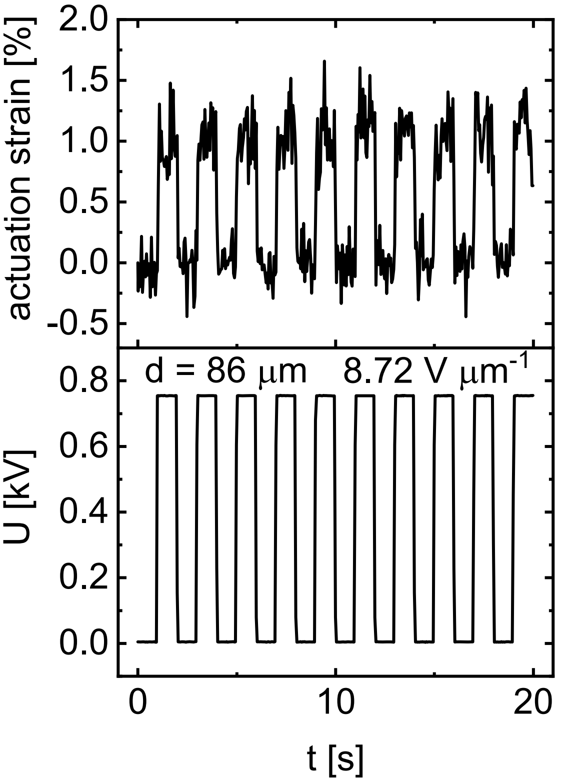

Figure S9. Cyclic tests of a single-layer actuator at different electric fields with a frequency of 1 Hz. At an electric field of 8.72 V μm^{-1} the actuation strain is 1.25 %.

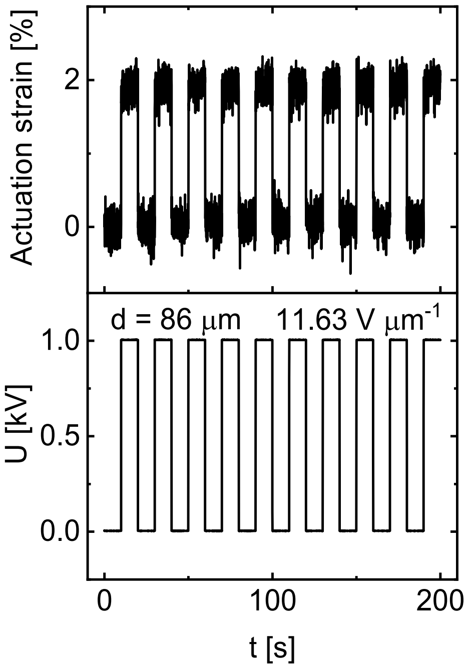

Figure S10. Cyclic tests of a single-layer actuator at different electric fields with a frequency of 100 mHz. At an electric field of 11.63 V μm^{-1} the actuation strain is 2.2 %.

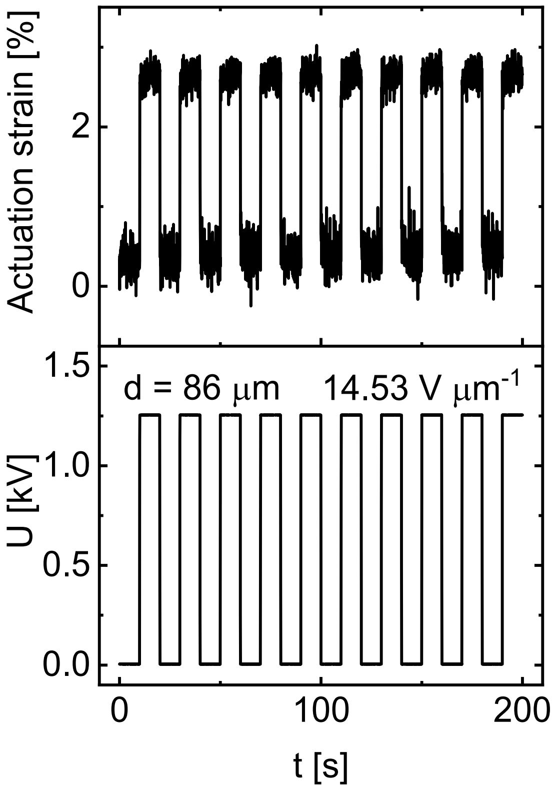

Figure S11. Cyclic tests of a single-layer actuator at different electric fields with a frequency of 100 mHz. At an electric field of 14.53 V μm^{-1} an actuation strain of 2.8 % is reached.

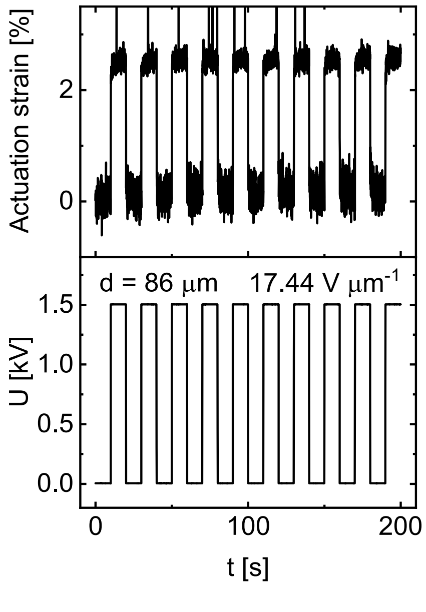

Figure S12. Cyclic tests of a single-layer actuator at different electric fields with a frequency of 100 mHz. At an electric field of 17.44 V μm^{-1} an actuation strain of 2.8 % is reached.

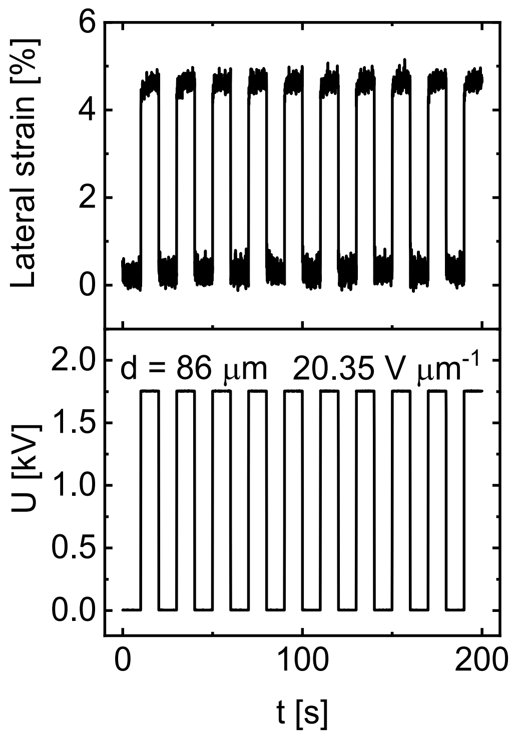

Figure S13. Cyclic tests of a single-layer actuator at different electric fields with a frequency of 100 mHz. At an electric field of 20.35 V μm^{-1} an actuation strain of 4.8 % is reached.

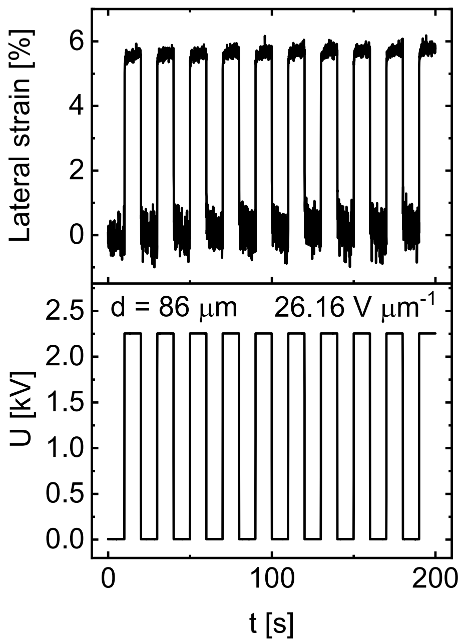

Figure S14. Cyclic tests of a single-layer actuator at an electric field of 26.16 V μm^{-1} with a frequency of 100 mHz show an actuation strain of 5.7 %.

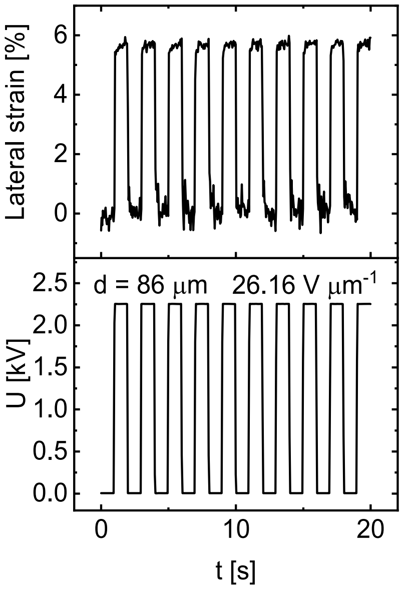

Figure S15. Cyclic tests of a single-layer actuator at an electric field of 26.16 V μm^{-1} with a frequency of 1 Hz show an actuation strain of 5.7 %.

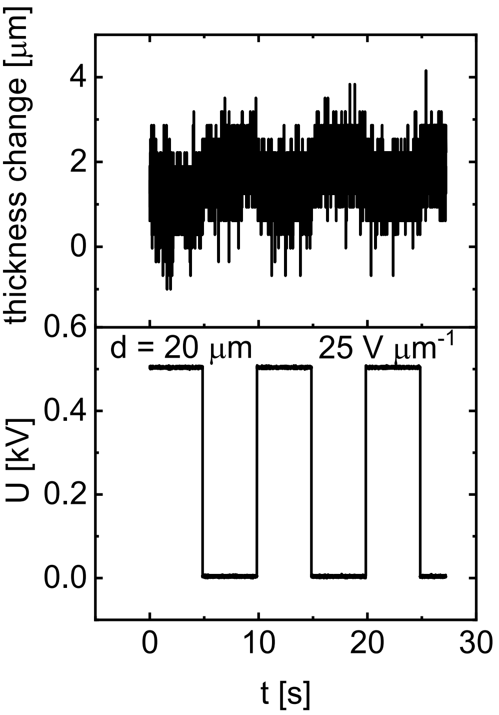

Figure S16. Screening of individual stack actuators at 500 V (25 V μm^{-1}) with a frequency of 100 mHz. Typical plot of an actuator with no actuation.

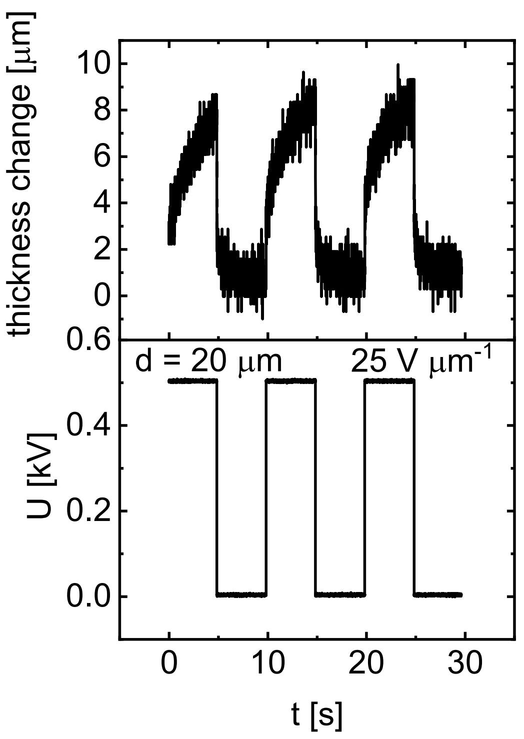

Figure S17. Screening of individual stack actuators at 500 V (25 V μm^{-1}) with a frequency of 100 mHz. Typical plot of an actuator with actuation between 0 - 10 μm.

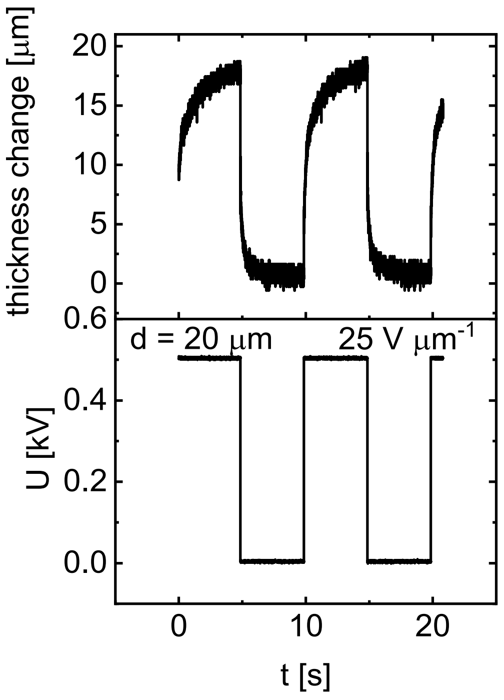

Figure S18. Screening of individual stack actuators at 500 V (25 V μm^{-1}) with a frequency of 100 mHz. Typical plot of an actuator with actuation between 11 - 20 μm.

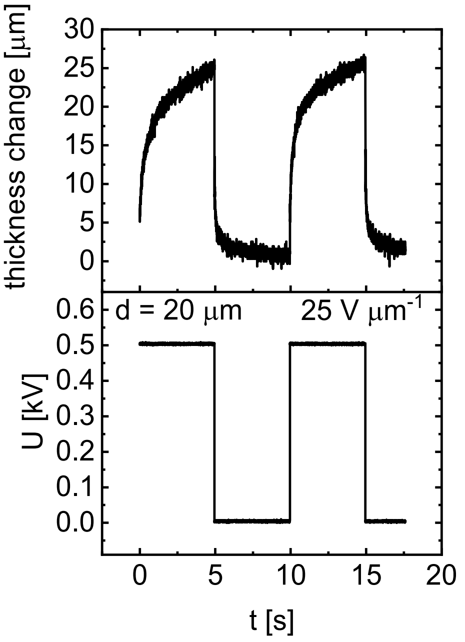

Figure S19. Screening of individual stack actuators at 500 V (25 V μm^{-1}) with a frequency of 100 mHz. Typical plot of an actuator with actuation between 21 - 30 μm.

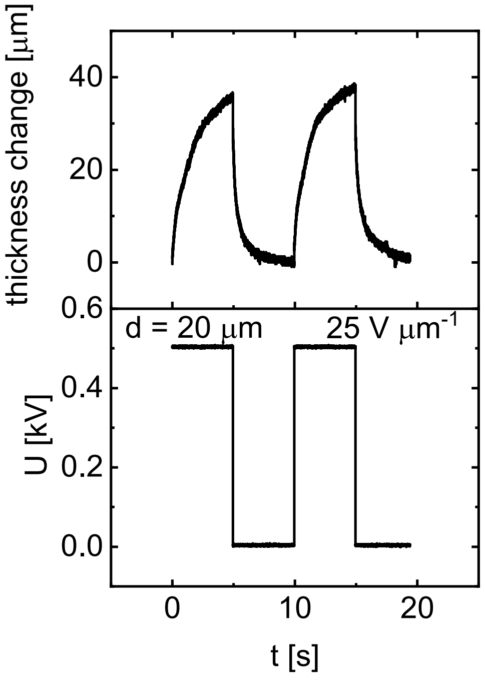

Figure S20. Screening of individual stack actuators at 500 V (25 V μm^{-1}) with a frequency of 100 mHz. Typical plot of an actuator with actuation between 31 - 40 μm.

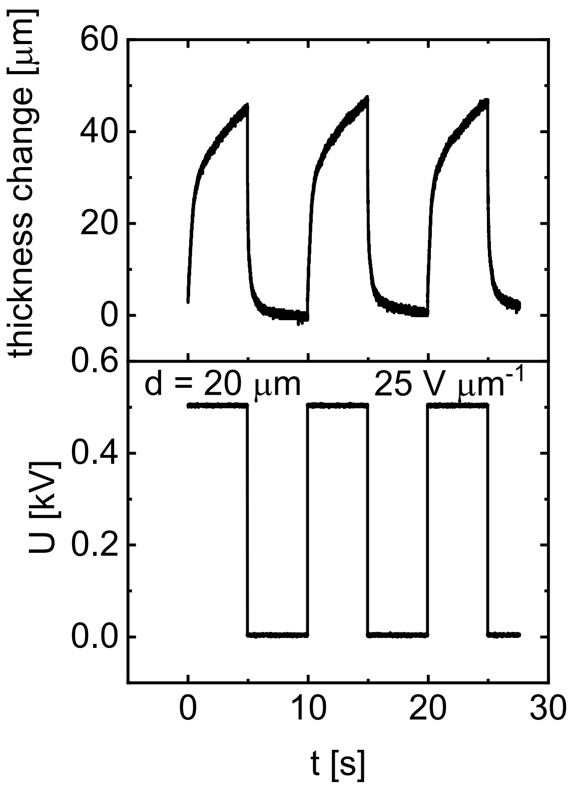

Figure S21. Screening of individual stack actuators at 500 V (25 V µm^{-1}) with a frequency of 100 mHz. Typical plot of an actuator with actuation between 41 - 50 µm.

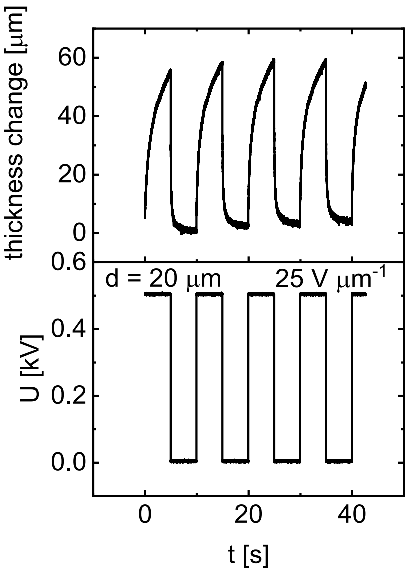

Figure S22. Screening of individual stack actuators at 500 V (25 V µm^{-1}) with a frequency of 100 mHz. Typical plot of an actuator with actuation between 51 - 60 µm.

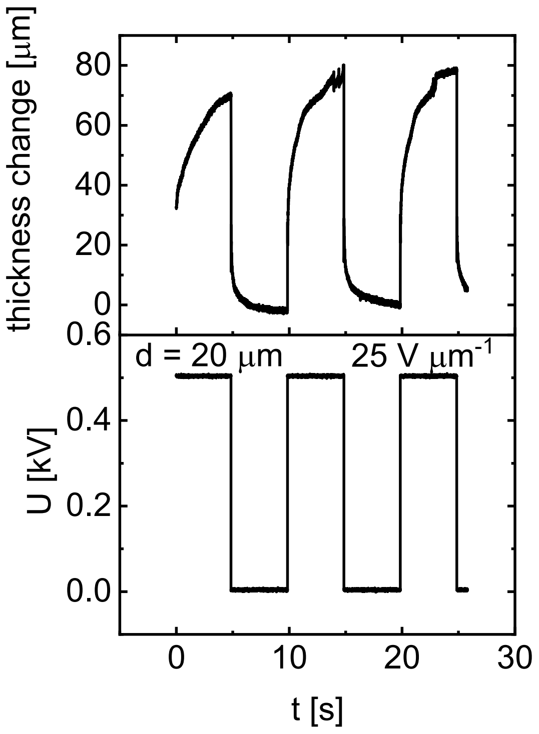

Figure S23. Screening of individual stack actuators at 500 V (25 V µm^{-1}) with a frequency of 100 mHz. Typical plot of an actuator with actuation above 61 µm.

Table S1. Comparison of material properties and linear actuation performance of state-of-the-art stack actuators.

Name	Permittivity	Young's Modulus	Electric field	Actuation strain
**This work**	11	0.35 MPa	25 V µm^{-1}	6.25%
**CT-SDEA**[3]	3	0.87 MPa	49.2 V µm^{-1}	3.3%
**PUR**[4]	7	3 MPa	50 V µm^{-1}	3.5%
**4N**[4]	2.8	1.1 MPa	50 V µm^{-1}	3.5%
**Printed stacks**[5]	11.39	0.50 MPa	17 V µm^{-1}	2.0%
**PDMS Sulfonyl**[6]	15	0.75 MPa	13.8 V µm^{-1}	2.5%
**PDMS chloro**[7]	6.4	0.577 MPa	12.4 V µm^{-1}	1.0%
**(MWCNT-Ecoflex)/Ecoflex**[8]	7.34	0.058 MPa	5 V µm^{-1}	7.11%
**Wacker P7670**[9]	3	0.5 MPa	50 V µm^{-1}	5%
**Sylgard 186**[10]	3	0.5 MPa	96 V µm^{-1}	4.7%

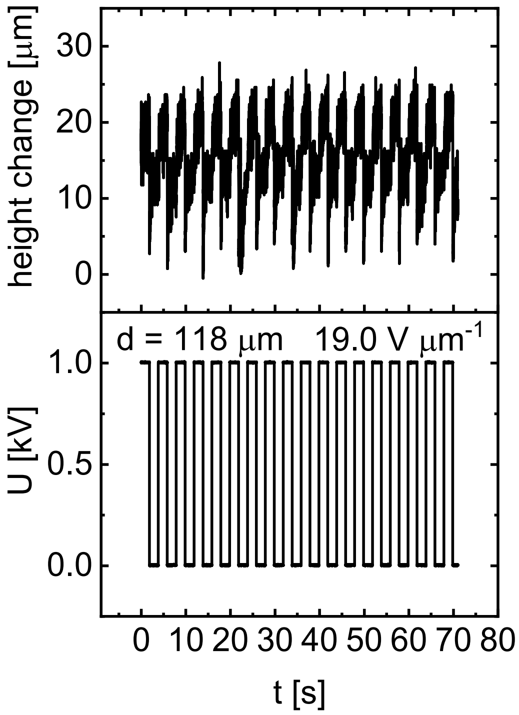

Figure S24. An actuator tested at 1000 V (50 V μm^{-1}) with a frequency of 250 mHz shows 26 μm height change.

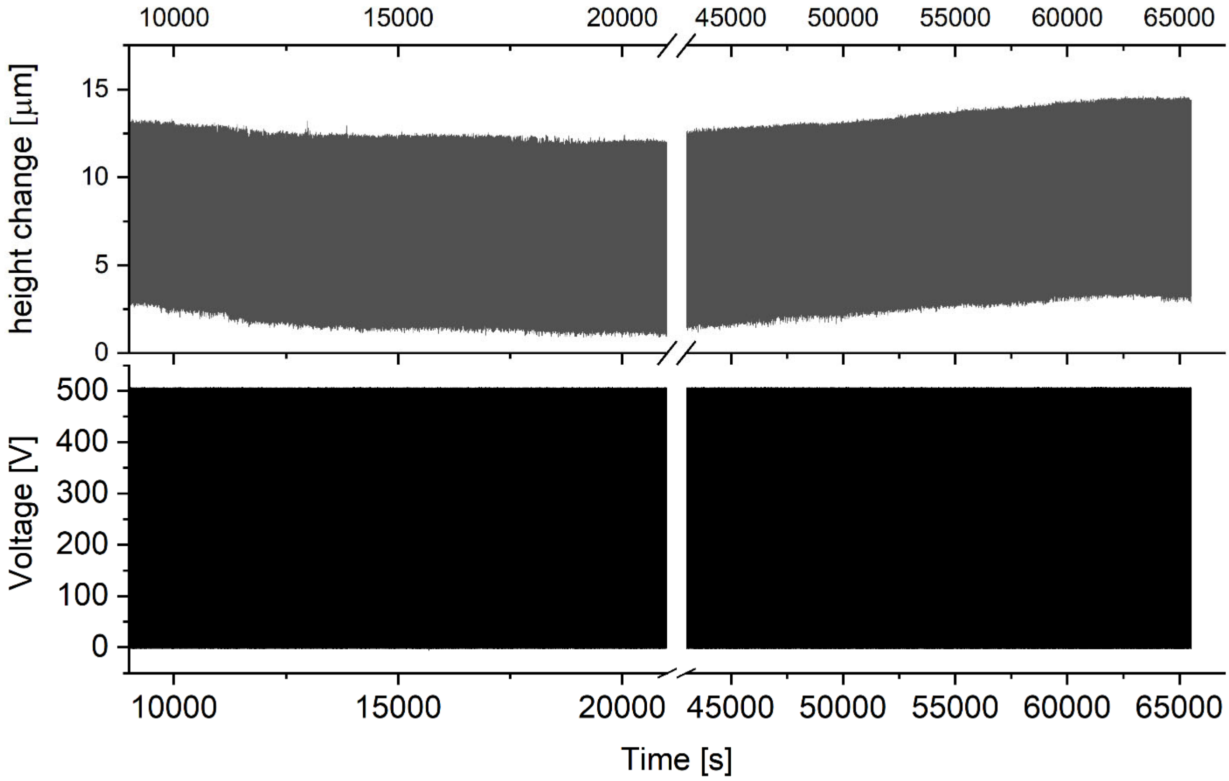

Figure S25. An actuator tested at 500 V (25 V μm^{-1}) at 250 mHz for over 16500 cycles shows a stable 12 μm height change.

1 J. van Turnhout and M. Wübbenhorst, *J. Non. Cryst. Solids*, 2002, **305**, 50–58.

2 M. Wübbenhorst and J. van Turnhout, *J. Non. Cryst. Solids*, 2002, **305**, 40–49.

3 A. Behboodi and S. C. K. Lee, *IEEE Int. Conf. Rehabil. Robot.*, 2019, **2019**-**June**, 499–505.

4 J. Maas, D. Tepel and T. Hoffstadt, *Meccanica*, 2015, **50**, 2839–2854.

5 P. M. Danner, T. Pleij, G. Siqueira, A. V. Bayles, T. R. Venkatesan, J. Vermant and D. M. Opris, *Adv. Funct. Mater.*, 2023, **34**, 2313167.

6 C. Zeytun Karaman, T. Raman Venkatesan, J. von Szczepanski, F. A. Nüesch and D. Opris, *J. Mater. Chem. C*, 2025, **13**, 15886–15896.

7 J. von Szczepanski and D. M. Opris, *Adv. Mater. Technol.*, 2023, **8**, 2201372.

8 X. Zhang, Z. Zhao, Z. Wang, W. Xu, K. Ke, Z. Liu and W. Yang, *ACS Omega*, 2025, **10**, 27127–27136.

9 H. Haus, M. Matysek, H. Mößinger and H. F. Schlaak, *Smart Mater. Struct.*, 2013, **22**, 104009.

10 S. Akbari, S. Rosset and H. R. Shea, *Appl. Phys. Lett.*, 2013, **102**, 071906.
